# A Review of Probabilistic Genotyping Systems: *EuroForMix*, *DNAStatistX* and *STRmix™*

**DOI:** 10.3390/genes12101559

**Published:** 2021-09-30

**Authors:** Peter Gill, Corina Benschop, John Buckleton, Øyvind Bleka, Duncan Taylor

**Affiliations:** 1 Forensic Genetics Research Group, Department of Forensic Sciences, Oslo University Hospital, 0372 Oslo, Norway; oeyble@ous-hf.no; 2 Department of Forensic Medicine, Institute of Clinical Medicine, University of Oslo, 0315 Oslo, Norway; 3 Division of Biological Traces, Netherlands Forensic Institute, P.O. Box 24044, 2490 AA The Hague, The Netherlands; c.benschop@nfi.nl; 4 Department of Statistics, University of Auckland, Private Bag 92019, Auckland 1142, New Zealand; john.buckleton@esr.cri.nz; 5 Institute of Environmental Science and Research Limited, Private Bag 92021, Auckland 1142, New Zealand; 6 Forensic Science SA, GPO Box 2790, Adelaide, SA 5001, Australia; Duncan.Taylor@sa.gov.au; 7 School of Biological Sciences, Flinders University, GPO Box 2100, Adelaide, SA 5001, Australia

**Keywords:** probabilistic genotyping, *EuroForMix*, *DNAStatistX*, *STRmix* ^TM^

## Abstract

Probabilistic genotyping has become widespread. *EuroForMix and DNAStatistX* are both based upon maximum likelihood estimation using a γ model, whereas *STRmix™* is a Bayesian approach that specifies prior distributions on the unknown model parameters. A general overview is provided of the historical development of probabilistic genotyping. Some general principles of interpretation are described, including: the application to investigative vs. evaluative reporting; detection of contamination events; inter and intra laboratory studies; numbers of contributors; proposition setting and validation of software and its performance. This is followed by details of the evolution, utility, practice and adoption of the software discussed.

## 1. Introduction

The use of software to evaluate DNA profile evidence is widespread in the forensic biology community. Since the late 1990 s software tools have been used to apply statistical evaluation models to observed DNA profile data. There are currently over a dozen different software applications that undertake this task. These can be grouped under the umbrella term ‘probabilistic genotyping’ (PG) systems. All evaluate DNA profile data within a probabilistic framework and provide a likelihood ratio (*LR*) to express the weight of evidence. The *LR* is the probability of the observed DNA profile data, given two competing propositions. Specifically, in the evaluation of DNA profile data within this framework, the *LR* is the ratio of the sum of weighted genotype sets that apply under each proposition.

In Section 2 we discuss some general, software-agnostic aspects of PG. We give an overview of available PG software and the class of modelling that each applies to carry out evaluation. An important aspect of any evaluation is the sensitivity of the *LR* to the data used to inform the model, and to the model choice itself (along with inherent underlying assumptions). Ideally, the *LR* would remain relatively stable regardless of the choices made within or between software (and therefore between models). There have been a number of validations for software individually, but also between laboratories using the same software, and between different software programs (Section 2.4 and Section 3.6). User inputs are important to deal with uncertainty about the number of contributors to a DNA profile and to define propositions that are most appropriate to evaluate the value of the evidence.

In Section 3 and Section 4 we review in detail three software applications; *EuroForMix*, *DNAStatistX* (these software utilise the same theory but have been independently prepared) and *STRmix™*. All are in regular use in multiple forensic biology laboratories around the world. These software applications utilise different models to describe DNA profile behaviour and have developed niche capabilities. There are also a number of support products, described in Section 3 and Section 4, that add functionality for the user, either to perform additional analyses, or to display results in an interactive or more intuitive manner. 

## 2. Probabilistic Genotyping in Generality

### 2.1. Probabilistic Genotyping Software

The recommended method for evaluation of DNA profile data in the forensic field is the *LR* [1,2,3]. It is assumed that autosomal markers are independent and in Hardy–Weinberg equilibrium. The *LR* seeks to determine the probability of obtaining some observed data (*O*) given a pair of competing propositions (*H*_1_ and *H*_2_), and any background information (*I*) about the framework of circumstances of the case that is relevant to the evaluation. Formulaically, the *LR* is expressed as:

(1) $$LR=\frac{Pr(O|H_{1},I)}{Pr(O|H_{2},I)}$$

From this point on we omit the background information term, *I*, for visual clarity but note that it is ever present in the evaluation of any data. To calculate the *LR*, as shown in Equation (1), a number of nuisance parameters must be considered. The most fundamental of these (universal to any method of *LR* assignment) is the set of genotypes, *S*, that could belong to individuals whose DNA is present in the profile. Incorporating the *J* possible genotype sets into the *LR* from Equation (1) gives:

(2) $$LR=\frac{\sum_{j=1}^{J}Pr(O|S_{j})Pr(S_{j}|H_{1})}{\sum_{j=1}^{J}Pr(O|S_{j})Pr(S_{j}|H_{2})}$$

The terms $Pr(S_{j}|H_{x})$, $x\in \left\{1,2\right\}$, refer to the prior probability of observing the genotype set given a proposition. If the proposition specifies the presence of a particular individual, then any genotype set that does not contain the genotype corresponding to that individual (and depending on the model, the genotype of that individual in a specific component of the evidence profile) necessarily has a probability of 0. Any other genotype set has a prior probability that is assigned based on population genetic models and allele frequency databases. The terms $Pr(O|S_{j})$ in Equation (2) are the probability of obtaining the observed data given a particular genotype set. These are often referred to as weights (and given the short-hand nomenclature of $w_{j}$) and are independent of propositions. The assignment of weights in the *LR* has been fundamental to much of the advancement that has occurred in probabilistic genotyping software used to interpret mixtures can be divided into three different groups;

Binary models

Qualitative, discrete or semi-continuous models

Quantitative, or continuous models

In early statistical models referred to as ‘binary models’, in which drop-out and drop-in were not considered, the weights were assigned values of 0 or 1, based on whether the genotype set accounted for the observed peaks (unconstrained combinatorial) and optionally on whether the peak balances were acceptable (constrained combinatorial). In essence binary models make yes/no decisions to associate genotypes with contributors, e.g., see the Clayton guidelines [4]. These early models were the precursors of more sophisticated methods that were introduced in later years. Whilst they perform calculations within a probabilistic framework, they are not probabilistic genotyping systems in nature as they do not treat the DNA profile information probabilistically, beyond specifying genotypes as being possible or impossible.

Later models referred to as qualitative (‘discrete’ or ‘semi-continuous’) [5,6,7,8,9,10] calculated weights as combinations of probabilities of drop-out and drop-in as required by the genotype set under consideration to describe the observed data. The qualitative models did not model peak heights directly but could use them to inform the nuisance parameter for the probability of drop-out or to infer a major donor genotype by applying different drop-out probabilities per contributor [11]. Whilst qualitative models do not use peaks heights directly, these systems do represent an advance over the binary model as they can take account of multiple contributors, low-template DNA and replicated samples.

Quantitative (or ‘continuous’) models [12,13,14,15,16,17,18] are the most complete because they take full account of the peak height information in order to assign numerical values to the weights. Using various statistical models these quantitative systems describe the expectation of peak behaviour in DNA profiles through a series of nuisance parameters that align with real-world properties such as DNA amount, DNA degradation, etc. A list of currently used PG software is provided in Supplement S1.

### 2.2. Investigative vs. Evaluative Forensic Genetics

The forensic scientist has a dual role as investigator and evaluator [3]. In conventional casework, a suspect is identified; the case-circumstances are reviewed, then the alternate propositions are formulated. This forms the basis of the court-case that the scientist will provide testimony. He/she is said to be in “evaluative mode” and the principles of interpretation apply as described, for example, by the ENFSI guideline [19]. 

Alternatively, a piece of evidence may be retrieved from a crime-scene, but there may not be a suspect available. In this instance the scientists will work in “investigation mode”. To identify potential suspects for further investigation a national DNA database is typically searched. 

Conventional database searches are usually restricted to searches of the person of interest (POI) from a crime-stain profile that has been deconvolved. This strategy is sufficient for single profiles and major/minor mixtures where the POI is represented in the former. However, if allele dropout has occurred and there are multiple contributors, then the POI may not be unambiguously resolved. The search is much more difficult, as many more candidates are possible, and it becomes much less likely to identify ‘true-donor’ candidates and more likely to obtain a long list of adventitious matches.

Probabilistic genotyping offers a much more complete way to search large databases. With a database of *N* individuals, each is considered as a possible candidate that is compared to the crime stain *O.* Consequently, a likelihood ratio can be generated for every individual in the database, where the propositions are:

*H*_1_: Candidate *n* is a contributor to the evidence profile *O*

*H*_2_: An unknown person is a contributor to the evidence profile *O*

Where all contributors to the profile not being considered as the candidate are designated as unknown and unrelated to the candidate. Consequently, for a well-represented DNA profile, the majority of candidates will return a low *LR* < 1, which means that they will be eliminated from the investigation; one or more may return *LR* > 1, and they are forwarded to the prosecuting authorities for further investigation. If the crime-stain is a low-template mixture of several contributors, the *LR*s will be lower and there may be numerous potential candidates, especially with searches of large databases of several million individuals. A list, ranked according to high→low *LR*, can be provided to investigators, but the extent of the investigation will be dependent upon the resources available. Lists may be shortened by prioritising candidates from a geographical location, or with known *modus operandi*. Once suspects are identified, they may become defendants and the scientist returns to evaluative mode reporting.

With complex cases, it may be of interest to identify individuals that may have contributed to multiple crime-stains. *STRmix™* utilises the semi-continuous method of Slooten [20] to compare the alternative propositions:

The DNA profiles have a common contributor

The DNA profiles do not have any common contributors (it is assumed that contributors are unrelated)

The method does not depend upon a database search or direct reference profile comparison.

*CaseSolver* [21] is based upon *EuroForMix* and is designed to process complex cases with many reference samples and crime-stains. Here, mixtures are compared against reference samples only—however, mixtures can be deconvolved so that unknown contributors found in other samples may be cross-compared. *SmartRank* [22,23] (qualitative) and *DNAmatch2* [24] (quantitative) are used to search large databases and can also be used in contamination searches.

### 2.3. Probabilistic Genotyping to Detect Contamination Events

Investigative searches extend to comparisons of samples to detect potential contamination events [25,26] that may be propagated either by:

Contamination of reagents or consumables by laboratory staff or other laboratory employees, or at the crime scene, or in the examination room by investigators.

Sample to sample cross contamination during processing.

Type 1 contamination may be detected if each sample/mixture is compared to an elimination database of, e.g., crime scene investigators and laboratory staff.

Type 2 cross contamination, e.g., between capillary electrophoresis (CE) plates may occur. An extreme example is illustrated by the case of “wrongful arrest of Adam Scott” [27] pp. 21–31, where CE plates were accidentally reused by the laboratory. However, the biggest risk is with accidental carry-over of DNA on reusable tips or by capillary carry-over, where PCR products injected by a capillary are not completely removed during the cleaning process [25].

In much the same way that the improvement in PG systems has led to an increased ability to identify donors to profiles in a criminal context, so too has the power improved to identify contamination events. Additionally, with the continual drive for high-throughput capabilities, many contamination searching processes within PG systems are either automated, part of the laboratories information management system, or able to be set-up and run in bulk with minimal human effort. For further details about investigative searches with *STRmix^TM^* refer to Section 4.5. *CaseSolver*, *DNAmatch2* and *SmartRank* are described in Section 3.4.

### 2.4. Inter and Intra-Laboratory Studies

The ultimate endpoint to a forensic biology evaluation is evidence presented in court. An expectation exists that information presented is reliable; one component of demonstrating reliability of PG systems, is to carry out studies on their practical use in casework. These studies can describe the performance of the PG systems in general (further details provided in Section 2.7), but also the consistency of their use in multiple laboratories by multiple people. Both inter- and intra-laboratory studies involve the distribution of mixtures with known ground truth, usually as electronic files after analysis, among forensic scientists within a laboratory and/or to a number of different laboratories. The compiled results give a measure of the variability in performance within and between laboratories [28,29,30,31,32,33,34,35,36,37]. At least two studies [38,39] (hereafter the GHEP-ISFG study and NIST studies) have appeared in courtroom discussion due to the wide range of results observed. 

The GHEP-ISFG study applies various PG software to the same mixture and has been discussed in admissibility hearings. The results using *LRmix* varied from 2.6 × 10^3^ to 3.2 × 10^14^. This variability is based primarily upon human decision making and interpretation, e.g., choice of drop-in probability; drop-out probability and sub structuring population correction. It is further aggravated by the presence of three pairs of unresolved peaks. The variation is not intrinsic to the software but does emphasise that high reproducibility will only come by carefully considering the human element. We also note that much of the variation in human decision making comes from different actions intended to be conservative. In other studies using *LRmix*, such as [40], the results are comparable.

The NIST studies predate PG but have been subsequently reworked [41] using *STRmix™*, *EuroForMix* v1.10.0, *EuroForMix* v1.11.4, *Lab Retriever*, *LRmix*, and RMP (random match probability) [42]. The quantitative software, *STRmix* and *EuroForMix* (both versions), produced similar results with the exception of ref 5C for case 5. The qualitative software, *Lab Retriever* and *LRmix*, also produced results similar to each other. RMP was given as a benchmark. 

Alladio et al. [43] compared *Lab Retriever*, *LRmix Studio*, DNA-VIEW^®^, *EuroForMix*, and *STRmix^TM^*. In general, the quantitative software DNA-VIEW^®^, *EuroForMix*, and *STRmix^TM^* performed similarly and the qualitative software *Lab Retriever* and *LRmix Studio* also performed similarly to each other, but differed from the quantitative methods. Alladio et al. concluded “*results provided by fully-continuous models proved similar and convergent to one another, with slightly higher within-software differences (i.e., approximatively 3–4 degrees of magnitude)*”. Iyer [44] has appealed to the community not to overlook the differences between software of the order of 3–4 orders of magnitude even in a pattern of overall similarity arguing that in some circumstances such differences could be crucial.

Alladio et al. suggested the use of a “statistic consensus approach [45]” which “consists in comparing likelihood ratio values provided by different software and, only if results turn out to be convergent, the most conservative likelihood ratio value is finally reported. On the contrary, if likelihood ratio results are not convergent, DNA interpretation is considered inconclusive.” In the paper, convergent (a) and non-convergent (b) are defined as the two results both having (a) LR > 1 or LR < 1 and (b) one result LR > 1 and the other is LR < 1. Using such an approach would deem ref 5C for case 5 of the NIST study inconclusive using EuroForMix (LR about 10^3^–10^6^) and STRmix (LR about 0). The ground truth is that ref 5C is a non-donor, although it was an artificial construct based on resampling alleles from the profile [41] and consequently represents an outcome that would be rarely observed in actual case-work. However, from a recent collaborative study [46] we note that STRmix is more likely to report lower LRs when the alternative contributor has a high degree of shared alleles (as in cases of relatedness). In a much-discussed case in upstate New York (NY v Hillary) the result would also have been reported as inconclusive (STRmix LR about 10^5^, TrueAllele LR not known but plausibly slightly less than 1). The ground truth in NY v Hillary is, of course, not known. Taylor et al. [47] take up the subject of the “statistic consensus approach” pointing out that either two quantitative or two qualitative systems should be used (this plausibly is also Alladio et al.’s view) and averaging might be better than taking the lowest. Furthermore, there is no particular reason to choose LR = 1 as a value to use in the definition of non-convergent. In fact, an LR that is the inverse of the, unknown, prior odds is more crucial from a decision theory perspective. To illustrate, suppose that the prior odds are 1:X, then it is not until the LR reaches X:1 that the posterior odds will begin to support a proposition that is potentially different from that supported by the prior odds. From a decision theory perspective, this is a threshold at which a switch may occur between two possible actions when making a decision. 

Swaminathan et al. [48] create four variants of their *CEESIt* software and note some large differences in the resulting *LR*. This is relatively unsurprising as the underlying differences between their four versions are quite substantial and they analyse very low peak heights. For example, one large *LR* difference is driven by a peak at 6 rfu.

Whilst the “*statistic consensus approach*” is a rational approach to lack of consistency between different software we would add that it is vital to increase efforts to diagnose, and hopefully remedy, the inconsistency. It is a great pity that much larger efforts have not been made in this regard. Some of the authors are currently involved in such an exercise and results are already greatly promising.

**Figure 1:**
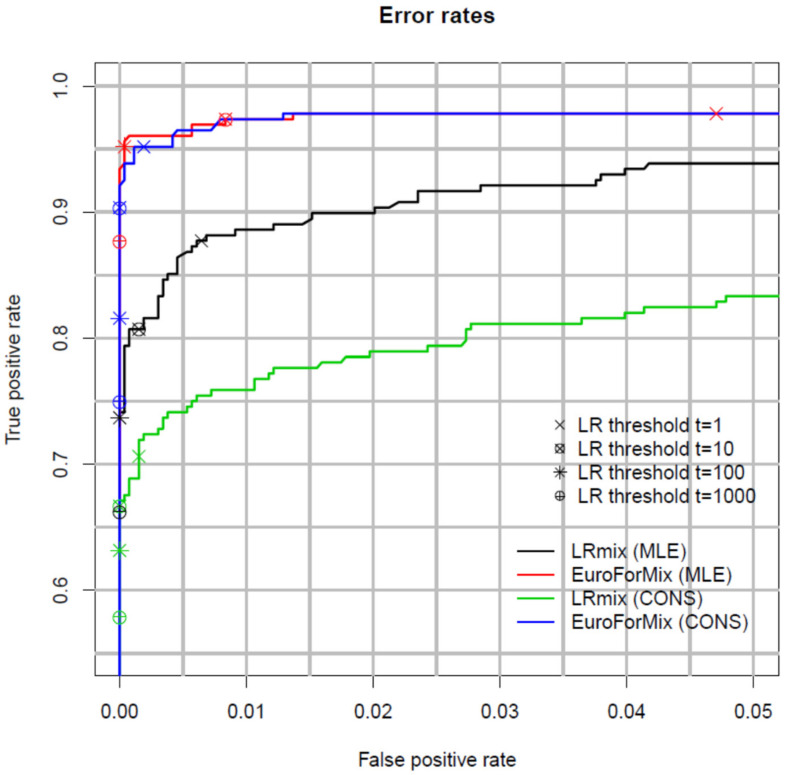
Receiver operating characteristic (ROC) plot where the rate of false positive support (FP) (horizontal axis) and true positives support (TP) (vertical axis) are plotted as a function of LR thresholds. The plot shows the results for the maximum likelihood estimation method (MLE) and the conservative method (CONS) for both *LRmix* and *EuroForMix.* The points on the curves show the FP and TP rates for different *LR* thresholds. Note that with this dataset, approximately 5% of samples were very low-level mixtures so that the POI was undetectable This caused the number of contributors to be underestimated, leading to very low (exclusionary) *LR*s. Therefore, the true positive rate does not reach 1.0 with the MLE method. Reprinted from [50], Copyright (2016), with permission from Elsevier.

A useful way to measure and compare the performance of models is with Receiver Operator Characteristics (ROC) plots [49]. These plots compare false positive support vs. false negative support rates relative to the observed *LR* (Figure 1). A good model simultaneously minimises the number of false positive and negative support for low values of *LR*. Figure 1 shows that the *LRmix* MLE and conservative qualitative models have lower true positive support rates compared to the quantitative *EuroForMix* MLE and conservative models, whereas false positive support rates are similar. This shows that the analysed quantitative models are more efficient; as discussed in the previous paragraph, this would not support a consensus approach between different classes (quantitative vs. qualitative) of models. For a given set of data, ROC plots are useful to compare performance of different models.

You and Balding [51], also carried out ROC analysis to compare *EuroForMix* with *LikeLTD.* These are both γ models, with differing modelling assumptions; the overall results were similar. *LikeLTD* modelled forward *n* + 1 and complex *n −* 2 stutter and improvement was observed with some low template samples (since the version of *EuroForMix* used did not support these type of stutters). Manabe et al. [52] compared *Kongoh* with *EuroForMix*, both *γ* models, again finding strong similarities.

The first interlaboratory study with *STRmix* was reported by Cooper et al. [33]. In a subsequent enlargement of this exercise [53] two samples were examined. For one sample 176 responses were received with *LR*s ranging from 10^28.3^ to 10^29.4^. The bottom and top values were obtained by variation in human judgement elements such as dropping a locus (lowest *LR*) and a laboratory procedure that used a bespoke artefact handling process (top *LR*). For the 173 responses to the other sample, nine false exclusions were obtained by assigning numbers of contributors (NOC) as one fewer than the number used in construction of the sample. The remaining *LR*s reported varied from 10^4.3^ to 10^6.6^ with most of the variation attributable to *GeneMapper^®^* ID-X analysis settings used. 

McNevin et al. [54] describe such variation as “extreme sensitivity” and set an expectation of much greater reproducibility in the reported statistic. This echoes a call, by for example the UK Forensic Science Regulator (pers. comm.) to obtain similar results regardless of the laboratory where the case is submitted. This would be dependent upon human factors, laboratory policy, and elements outside the province of the software, as well as the theory and application of the software itself.

#### Non-Contributor Tests and Calibration of the LR

Ramos and Gonzalez-Rodriguez [55] introduced the concept of “calibration of the likelihood ratio”. Their purpose was to: “highlight that some desirable behaviour of *LR* values happens if they are well calibrated”, meaning that the behaviour of the software is consistent with the expectations of a predefined model. Calibration applies a much more rigorous criterion than Turing expectation: the rate of non-contributor inclusionary support is at most the reciprocal of the *LR*, i.e., Pr(*LR* > *x|H*_2_) ≤ 1/*x* [56]. Calibration tests that *LR*s of any given magnitude are occurring at the expected rate. It has been applied to *STRmix™* and *EuroForMix* [57,58].

It becomes increasingly difficult to test *LR*s as they become bigger as the number of samples needed becomes prohibitively large. Importance testing appears to be a remedy for this problem [59,60].

### 2.5. Number of Contributors (NOC) 

In casework the number of contributors is unknown. This also holds for many mock samples, especially where at least one donor has left no detectable signal. When a parameter is unknown it is very useful to treat it as a nuisance parameter. We discuss some recently developed methods based on this principle.

For many years the assigned NOC to a DNA profile has been estimated by applying the maximum allele count (MAC) approach, often tempered by a human examination of peak heights. This approach uses the locus exhibiting the largest number of alleles at a locus, divided by two and rounded up to the nearest whole number [4,61] and ([62] chapter 7) and SWGDAM interpretation guidelines [63]. This method equates the NOC with the *minimum* number of contributors.

With such a method, the true NOC is uncertain, especially with high order mixtures (three or more) and/or low levels of DNA [64,65,66]. It is difficult to refer to the true NOC even in mock samples, but we will define it here as the number of donors that have left some signal above the analytical threshold.

Under- or over-estimating the NOC can affect the weight of evidence [67] with qualitative models [35,68,69]. 

With quantitative models, underestimating usually, but not always, leads to false negative support for the lowest template contributor. Overestimating tends to produce false positive support for non-donors, usually at relatively low *LR*s. The larger template donors are much more stable with respect to different NOC [70,71,72,73]. 

In some cases it is only possible to interpret the major contributor(s) of the DNA mixture. If minor contributors are not of interest, the NOC can be based upon the former, and this helps to simplify the model [72,74]. 

Increasing the number of loci, using those with a higher discriminatory power, or massively parallel sequencing (MPS) data of STR loci, resulted in fewer misinterpretations of the NOC compared to the MAC method [75,76,77,78]. 

Alternative methods using the total number of alleles (total allele count, TAC), the distribution of allele counts over the loci, the population’s genotype frequencies, peak heights (PH), replicates, probability of allelic drop-out and stutter, or a Bayesian network approach have shown to yield improved NOC estimates [68,79,80,81,82,83,84,85,86,87,88,89]. 

The latest advances for estimating the NOC rely on machine learning approaches enabling optimal use of the available profile information. To date, a few models have been developed for use in forensic DNA casework [37,90,91,92]. These models make use of more information than the previously developed approaches since they are trained on a separate ground truth dataset. A big benefit of the machine learning approaches is that the estimation of the NOC can be performed in seconds, which is of importance in cases requiring rapid analyses. See Section 3.1.3 for a description of the NOC-tool used in *DNAxs*. The drawbacks of machine learning approaches are: (a) the requirement of large datasets that are specific to the laboratory that generated the data; (b) lack of transparency—the method of prediction may not be clear.

The need to assign the NOC for weight of evidence calculations is optimally treated by considering it as a nuisance parameter [71,92,93,94,95,96,97,98].

In an elegant mathematical development Slooten and Caliebe [94], making a few reasonable assumptions, show that the *LR* considering a reasonable range of NOC is the weighted average of the *LR* for each separate NOC. Specifically: $LR=\sum_{i}LR_{i}w_{i}\;$ where

$$LR_{i}=\frac{Pr(O_{c},O_{p}|H_{1},NOC=i)}{Pr(O_{c},O_{p}|H_{2},NOC=i)}$$

where $w_{i}=Pr(NOC=i|H_{2},O_{c},O_{p})$ terms the weights, and *O_c_* and *O_p_* are the genotype of the crime profile and the POI, respectively. The weights are the probability of the number of contributors given the profile and assuming the POI is not a donor. This is the term that has been assessed subjectively for many years and can now be assigned as a probability distribution, sometimes with the assistance of software.

In an alternative approach, used with *EuroForMix*, the effective NOC is decided by maximizing the likelihood adjusted by application of the ‘Akaike information criterion’ (AIC) [99], which favours simpler models to explain the evidence. The smallest number of unknown contributors needed to explain the evidence usually maximises the respective likelihoods.

These approaches can be very useful since it is not necessary to define an absolute NOC and the field should move this way, though most of the current probabilistic genotyping systems still require that the user specifies the NOC [100]. *STRmix™* v2.6 and higher treat NOC as a nuisance parameter but is currently only validated for taking into account two consecutive NOC values (say NOC = 3 or 4).

### 2.6. Proposition Setting/Hierarchy of Propositions

The application of Bayes’ rule in odds form requires at least two propositions which are usually chosen to align with the prosecution position based upon the case circumstances and a reasonable alternative. The alternative will also be based on the case circumstances, ideally on information given by the defence (thus, the alternative is often referred to in the literature as the defence proposition). 

There are at least two views of how the alternative should be set:

The scientist for the defence should assign this proposition, or in the absence of any meaningful consultation with the defense the scientist advising the prosecution assigns a reasonable alternative that is consistent with the best defense proposition and has a good approximation to exhaustiveness.

The concept of the hierarchy of propositions is well established [101,102]. Gittelson et al. [103] discussed this concept more recently; the ISFG DNA commission provides an extensive review [3], with recommendations for practitioners, also summarised by Gill et al. [62], chapter 12. 

Propositions are classified into four levels: offence, activity, source, and sub-source. 

Offence level propositions describe the issue for the fact finder which is one of guilt or innocence. This is a decision of the court; the forensic scientist does not offer opinions at this level.

Activity level propositions describe the activity that deposited the DNA. Provided that there is sufficient information, the forensic scientist may assist the court.

Source level refers to the origin of the body fluid or cell type examined. This is relatively straightforward if there is sufficient body fluid to test but may be challenging to address if there are low level mixtures of body-fluids.

Sub-source level refers to the origin of the DNA (i.e., donor). 

It has proven useful to use a fifth level. 

Sub-sub-source refers to the origin of part of the DNA, for example the major donor [104,105].

Probabilistic genotyping only provides information at sub-source and sub-sub-source levels. In order to make inferences at source and activity levels, separate calculations are required. If the distinction between levels in the hierarchy is not properly explained, it may lead to “carry-over” of the LR from one level to another which can lead to miscarriages of justice [3,27,106,107].

### 2.7. Validation of PG Systems

There are several publications that address ‘validation’ from scientific societies; for example: SWGDAM [108], ISFG [109], the AAFS Standards Board [110] and the UK Forensic Science Regulator [111]. Some laboratories have published validation studies—see Coble and Bright [100] for an excellent review and other guidance [108,109,112,113,114,115,116,117]. 

The purpose of validation is to define the scope and limitations of software. This is described in detail for *STRmix* (Section 4.6) and *EuroForMix*/*DNAStatistX* (Section 3.6) and Gill et al. [62] chapter 9.

George Box (a British statistician) famously stated: “Essentially, all models are wrong, but some are useful” [118]. All models are “wrong” in the sense that they are approximations of some unknown reality. However, so long as models demonstrate an empirical behaviour that conforms to expectations of a given reality, then they are “useful”. The question that follows in relation to different PG software is whether models that are based upon different theories and assumptions are “equally reliable” or “equally useful”?

The terms “right” or “wrong” are two extremes. Probability is a numerical description, somewhere between 0 and 1, which describes how likely it is that an event will occur. Importantly, probability represents a personal belief about uncertainty, that is informed by available data. Provided scientists use the same or similar datasets and the same methods of analysis, then their personal beliefs should coincide. We never know if something is true or not, but probability is always conditioned upon some hypothesis/ proposition being true.

As an example, consider the probability assigned for an allele that has never been seen before in the population sample (hereafter “rare allele”), but is observed in this case. We can say for certain that the “true” probability of this allele is not 0, but we are uncertain exactly what it is. Whenever something is unknown and uncertain it is best to model the uncertainty with a probability density function. A workable option may be to insert a reasonable point estimate. Further, in forensic science, some aspects of utility are usually confounded into the probability assignment by deliberately biassing the assignment in a direction thought to be conservative. However, in mixture evaluation the conservative direction is very uncertain. For example, it is typically conservative to raise the sample allele probability for the alleles that correspond with the person of interest (POI), but for any other alleles the effect may be neutral or may vary either way. The use of a point estimate biased upwards (for example 5/2*N* or 3/2*N* where *N* is the number of alleles in the sample) is plausibly conservative on average, although we are unaware of any systematic investigation of this assumption. The use of a probability density distribution and resampling may enable the choice of a conservative quantile but requires assignment of a distribution. It would be very difficult, and be a matter of subjective judgement, to choose which of these methods is appropriately conservative.

In the context of PG software, where two software may implement two different models for the same process we can assess how well the models describe the empirical data, then we can have confidence in the result. This can readily be supplemented by varying the model within reasonable limits dictated by the data and thus creating a range of plausible outcomes. We are left with the uncertainty that small modelling and inferential errors accrue, or that the training data for the models are inappropriate.

There are various phases to a validation programme, originally described by Rykiel [119] in relation to ecological models:

Conceptual validation: verification of the mathematical formulae used in the software are correct.

Software validation: Verification and testing of the code, e.g., by running test scripts.

Operational validation: The output of the model is tested against a wide range of evidence types, representing a typical case, as well as extreme examples.

A validation programme can address the following:

(a) Sensitivity (demonstrate the range of *LR*s that can be expected for true contributors)
(b) Specificity (demonstrate the range of *LR*s that can be expected for non-contributors)
(c) Precision (variation in *LR*s from repeated software analyses of the same input data)

Accuracy of statistical calculations and other results (comparison to an alternate statistical model or software program)

Determination of the limits of the software (either computational or conceptual, regarding for instance the number of unknown contributors or types of DNA profiles)

Steps towards internal validation, to enable a laboratory to adopt a given procedure, was described by [115] as an “accumulation of representative test data within the laboratory to demonstrate that the established parameters, software settings, formulae, algorithms and functions perform as expected” 

In real casework, we do not know the ground truth. In validation, the model is tested against samples where the ground truth is usually known. This enables two kinds of tests to be carried out using the standard likelihood ratio formula: *LR* = *Pr*(*O|H*_1_)/*Pr*(*O|H*_2_)

(a) *H*_1_ = true: where we know the POI is a contributor.
(b) *H*_2_ = true: where we know that the POI is not a contributor

As a small word of caution: the ground truth is not known even for mock samples for very low level contributors. For these it can be unclear whether they are, in reality, a donor at all.

## 3. Evolution of *EuroForMix* and *DNAStatistX*

### 3.1. Evolution

**Figure 2:**
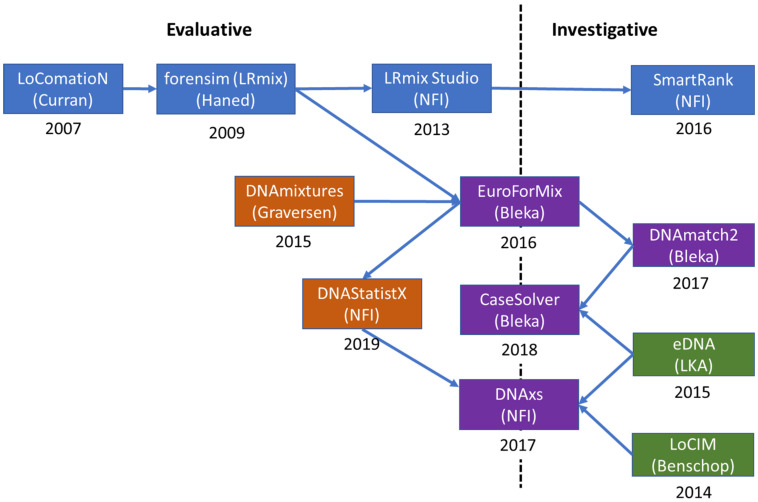
A diagram showing the evolution of probabilistic genotyping software developed by the NFI and Oslo University Hospital. Blue and orange boxes indicate qualitative and quantitative (γ) models, respectively. Green boxes are binary methods and purple boxes indicate software that include multiple types of methods.

An outline of the development and evolution of the software *EuroForMix* and *DNAStatistX*, including its predecessors and related modules is shown in Figure 2. These software will be discussed in the next sections.

#### 3.1.1. Qualitative Software

The development of probabilistic genotyping undertaken by the authors began in 2007 with the development of qualitative software (discrete or semi-continuous) which took account of allele drop-out and drop-in, but peak heights were not modelled. The first software was the introduction of *LoComatioN* by James Curran [120], whilst at the Forensic Science Service (UK). The model was re-programmed by Hinda Haned [121,122], as part of her PhD at the University of Lyon: *LRmix* is written in R and the module is found in the *forensim* package: https://forensim.r-forge.r-project.org/ accessed on 28 September 2021. Four years later, in 2013, the Netherlands Forensic Institute (NFI) adopted *LRmix*, rewriting the code into Java and rebranding it as *LRmix* Studio: https://github.com/smartrank/lrmixstudio accessed on 28 September 2021. This software has been widely adopted in Europe and elsewhere. LRmix Studio was further developed by NFI to provide SmartRank: https://github.com/smartrank/smartrank accessed on 28 September 2021, a database search engine [23] which was shown to be more efficacious than the CODIS search engine [22]; it is still widely used by caseworkers (see collaborative study of Prieto et al. [40]).

For further details see Gill et al. [62] chapters 5 and 6. Exercises and presentations are available from: https://sites.google.com/view/dnabook/chapter-6?authuser=0 accessed on 28 September 2021.

#### 3.1.2. Quantitative Software

Early models designed to explain variation in peak area observations were described in 1998 by *Evett* et al. [123] who defined an underlying normal distribution and in 2007 by *Cowell* et al. [18,124] who also defined a γ distribution (the γ model).

In 2013, Cowell, Graversen and colleagues released *DNAmixtures* which was based on the γ model [125,126]: http://dnamixtures.r-forge.r-project.org/ accessed on 28 September 2021, written in R code as open-source, but requires HUGIN (commercial software) to run it. Supported by the EU-funded EuroForGen-Network- of-Excellence: https://www.euroforgen.eu/ accessed on 28 September 2021, the γ model was re-written in R and C++ by Øyvind Bleka as *EuroForMix*: http://www.euroformix.com/ accessed on 28 September 2021. This program had enhanced capabilities compared to *DNAmixtures*, including degradation parameterisation and “theta-correction” (*Fst*).

*EuroForMix* was further utilized to provide the database search tool *DNAmatch2*, which also incorporated the *forensim LRmix* module, in order to carry out searches of large national DNA databases. Later, the same modules were integrated into a more user-friendly expert system called *CaseSolver* which is integrated into casework for analysing complex cases where there are multiple suspects and case-stains. *CaseSolver* includes many useful features for caseworkers: Visualization, automated comparison, deconvolution, weight-of-evidence evaluation and reporting (discussed in Section 3.4).

In 2019, the NFI implemented *DNAStatistX*, the statistical module based on the *EuroForMix* code which is further elucidated in Section 3.3.1. *DNAStatistX* can be used as a stand-alone application or within the DNA eXpert System, *DNAxs*. *DNAxs* is a software suite that was developed by the NFI, for the data management and (probabilistic) interpretation of DNA profiles. It was implemented in forensic casework in 2017 and is under continuous development to further advance the software, to improve the process of DNA casework and to broaden the scope of application. Further information on the *DNAxs* functionalities is provided in the following section.

#### 3.1.3. DNAxs and Related Modules

Increased complexity of DNA profile comparisons and interpretation demands fast and automated software tools to assist DNA experts in routine casework. *eDNA* is one such application [127], whose functionalities were an inspiration for the development of *CaseSolver* [21] and the *DNA eXpert System DNAxs* [128].

Within *DNAxs*, profile comparisons can be achieved at various levels:

(1) By aggregating replicate profiles into one composite view (bar graphs)
(2) By viewing the trace profile as bar graphs underneath which alleles of reference profiles are comparedThrough the match matrix option
(3) By sending a DNA profile for a *SmartRank* search against the DNA database
(4) By calculating *LR*s using *DNAStatistX* for a comparison of a person of interest to a trace DNA profile [128]

*DNAxs* imports (pre-analyzed) DNA profiling data which is shown as the original electropherogram and is graphically represented as bar graphs with a color coding for reproduced and non-reproduced alleles in case of PCR replicates, and a color coding for alleles of the major component of a mixture through the *LoCIM* method (Locus Classification and Inference of the Major) [29]. This *LoCIM* method can be applied to one amplification of a DNA extract or to replicate DNA profiles. In the latter case, *LoCIM* first generates a consensus profile that includes alleles that are observed in at least half of the replicates [86]. Next, *LoCIM* classifies each locus as type I, II or III based on thresholds for peak height; ratio of major to minor contributors; and heterozygote balance. A Type I locus fulfils the most stringent criteria and will most likely be correctly inferred. Type II loci may have lower peak heights or a smaller difference in peak heights compared to minor donors. Type III loci do not meet one or more of the Type II criteria and are the most complex to infer a major contributor’s genotype. Lastly, thresholds are used per locus type to infer the major component’s alleles. It has been demonstrated that the *LoCIM* approach is successful regardless of the laboratory’s STR typing kit and PCR and CE settings and the method is easy to implement (one only needs to specify the laboratory’s stochastic threshold) [29,37].

The major contributor’s genotype predicted by the deconvolution method of *EuroForMix* described in Section 3.3.4 (on loci with a probability that was at least twice as large as the second likeliest genotype possibility) was compared to that of *LoCIM* (on type I and II loci). Both methods are able to perform deconvolution by utilizing the peak height information, though *LoCIM* is threshold based while *EuroForMix* applies a statistical model which consists of a set of parameters which are inferred by maximizing the likelihood function [50]. *EuroForMix* applies a more comprehensive statistical model which calculates the uncertainty of different suggested genotype profiles extracted from the inferred uncertainty of the whole evidence profile. Therefore, these calculations are much more computationally intensive compared to the extremely fast *LoCIM* method. At the locus level, and as expected, the *EuroForMix* deconvolution showed improved performance compared to *LoCIM* [50]. Regardless, since *LoCIM* is extremely fast and was regarded useful to many cases, this approach was implemented in *DNAxs* [62], chapter 10 and [37]. 

*DNAxs* provides summary statistics for its comparisons, such as the number of mismatches or unseen alleles, and to help estimating the NOC- such as the maximum allele count (MAC) and the total allele count (TAC). Furthermore, *DNAxs* includes NOC tools based on a machine learning approach. These are designated as the RFC19 model that is specific to PowerPlex Fusion 6C (PPF6C) data as generated within NFI [89] and the generic RFC11 model which is laboratory independent [37]. The RFC19 model outperformed the MAC method and an in-house developed tool that utilised the TAC [89,91]. A drawback of such models is that it requires a large dataset for development and is specific to a laboratory’s data. To that end, the generic model was developed, which only involves features of the 12 European Standard Set and U.S. core loci, and does not include features holding information on peak heights or fragment lengths. The generic RFC11 model overall showed improved NOC estimates for data of different laboratories when compared to the MAC method but performed less efficiently when compared to the PPF6C specific RFC19 model, since it uses less of the available information. However, in absence of a data specific machine learning NOC model, or in absence of data or too limited resources to develop such model, the generic RFC11 model was found to be a useful alternative that can serve as an addition to the reporting officer’s toolbox to interpret mixed DNA profiles [37]. Another drawback of machine learning models is their lack of transparency; the model outputs a prediction but not how it obtained to the particular result. Therefore, in a study of Veldhuis et al. [129], eXplainable artificial intelligence (XAI) was introduced to help users understand why such predictions are made. 

Lastly, through web APIs (Application Programming Interfaces) *DNAxs* can communicate with, for instance, CODIS, LIMS systems, *SmartRank*, and *Bonaparte* [128]. Additionally, as previously mentioned, for weight of evidence calculations, *DNAxs* implements *DNAStatistX*, which, alike *EuroForMix,* uses the γ distribution to model peak heights. 

### 3.2. The γ Model 

The model adopted by the authors is known as the “γ model” which was first described by Cowell et al. [124,130]. 

The γ distribution is defined by two parameters known as shape *α* and scale *β*. There is a different shape parameter per contributor in the *EuroForMix* model, but there is only one (universal) scale parameter that is applied. The observed peak height is given as *y*.

The probability density function of the γ distribution is:

(3) $$f\left(y|\alpha ,\beta \right)=\frac{1}{\beta^{\alpha }\Gamma \left(\alpha \right)}y^{\alpha -1}\exp(-\frac{y}{\beta })=gamma\left(y|\alpha ,\beta \right)$$

where α and β are the shape and scale parameters, respectively, and Γ(*x*) is the γ function. The density function given in Equation (3) and provides the ‘weightings’ in *EuroForMix* and *DNAStatistX*.

The shape and scale parameters are calculated based on the following model parameters (for two donors):

*M_x_*: the mixture proportion for contributor 1 and *1-Mx,* the mixture proportion for contributor 2

*µ*: the peak height expectation (close to the average peak heights)

*ω*: the coefficient of peak height variation (indicates variability) 

**Figure 3:**
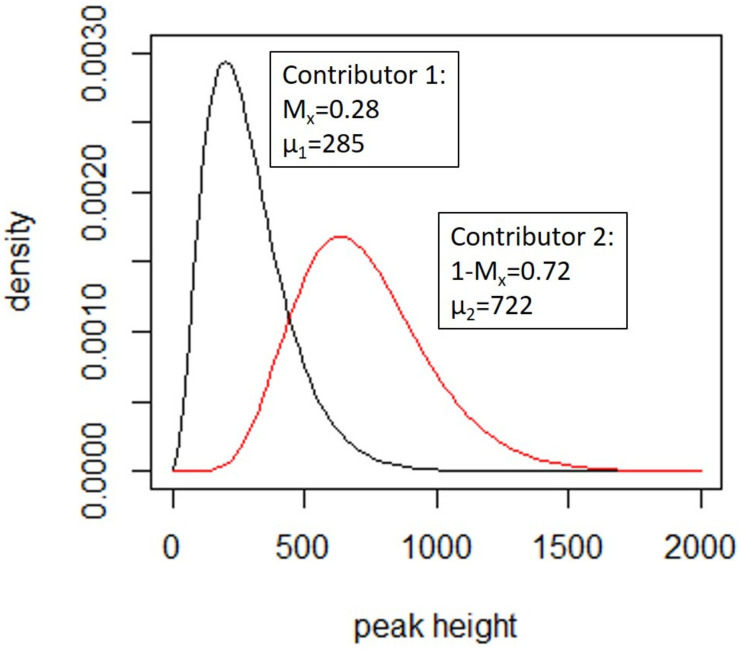
γ distributions for a simple case, where shape parameters = 3.312 and 8.381, respectively and the scale parameter is 86.2. The peak height expectation (*µ*) and *Mx* are shown for each contributor. The probability density function for the individual peak height contributions are derived from these curves. Reprinted from [62], chapter 7, Copyright (2020) with permission from Elsevier.

An example is provided in Figure 3. Further details are in Supplement S2.

There is a detailed explanation of the model, in Gill et al. [62], chapter 7. 

For a more detailed explanation, as applied to *EuroForMix* and *DNAStatistX,* see Gill et al. [62], chapter 7 and associated website where excel spreadsheets, tutorials and exercises can be downloaded: https://sites.google.com/view/dnabook/chapter-7?authuser=0 accessed on 28 September 2021.

The complexity of the γ model is increased by additional parameters: degradation, forward and backward stutter.

### 3.3. An Outline of the γ Model Incorporated into Euroformix and DNAStatistX

The aim is to quantify the value of evidence if a POI is a contributor to a crime-scene profile *O*. Two alternative propositions are specified and the likelihood ratio (*LR*) evaluates how many more times likely it is to observe the evidence given that *H*_1_ is true compared to the alternative that *H*_2_ is true.

#### 3.3.1. Model Features

*EuroForMix* and *DNAStatistX* support multiple contributors, can condition upon any number of reference profiles and can specify any number of unknown individuals, although there is a practical limit of c. 4 due to computational time.

1. The software accommodates degradation, allele drop-out, allele drop-in, *‘n −* 1’ and ‘*n* + 1’ stutters and sub-population structure (*Fst* correction). Note that stutters are not accommodated in the current version of *DNAStatistX*, but is under development for a future version.
2. Replicated samples can be analysed. Consensus or composite profiles, a feature of pre-PG software, are not used.
3. The model assumes same contributors and the same peak height properties for each replicate.
4. Optional Locus specific settings (*DNAStatistX* from v1, *EuroForMix* v3 onwards) are as follows:
  (a) Analytical threshold
  (b) Drop-in model
  (c) Fst correction

Although *EuroForMix* and *DNAStatistX* are based upon the same model, there are some differences. The software are programmed in different languages (*EuroForMix* in R and C++ and *DNAStatistX* in Java) and therefore not all of the numerical libraries *EuroForMix* uses were available when developing *DNAStatistX*. As a result, alternative methods for function optimization were explored and selected. Despite the differences in the choice of function optimizer, the two software yield *LR*s in the same order of magnitude when the same data and model options are used [128]. *DNAStatistX* is implemented within the overall software package, *DNAxs,* which supports parallel computing that can be delegated to a computer cluster and enables queuing of requested *LR* calculations. This feature can be extremely useful in a routine casework setting. Both software continue development though functionalities and options can be prioritized differently by their developers and users.

Whereas *DNAxs* parallelises over independent function optimizations (current version), *EuroForMix* applies parallelisation within the inner part of the algorithm, where genotype summation is performed (versions before v3 also parallelised over function optimizations).

#### 3.3.2. Exploratory Data Analysis

The reported *LR* is critically dependent upon the assumptions applied in the model. The parameters that are fixed include: the population database including allele frequencies, the level of *Fst* and the drop-in parameters used to specify the drop-in model. 

The variable parameters are mixture proportions (Mx), peak height variation (coefficient-of-variation), peak height expectation and the NOC. Decisions are needed whether to use a stutter and/or a degradation model: Real case examples typically employ degraded DNA causing a reduction in observed peak heights when the molecular fragment lengths increase. The stutter models are important to apply when stutter filters are not applied—nevertheless there may still be alleles present in the profile which could be explained as stutters. In addition, the number of contributors can have an impact—so this must be carefully decided (Section 2.5).

Finally, any model that is used for reporting must be a reasonable fit to the γ distribution. In order to highlight the principles of exploratory data analysis, details are described by Gill et al. [62] (chapter 8). 

#### 3.3.3. Relatedness

The defence may wish to put forward a proposition that a sibling (or another close relative) was the contributor to the crime stain, hence the defence alternative considered may be *H*_2_: “The DNA is from a sibling of Mr. X”.

The calculations are described using formulae described by Gill et al. [62], chapter 5.5.4 and appendix A.2; encoded into *LRmix Studio* and *EuroForMix*. Examples can be found from the “Relatedness” folder at: https://www.dropbox.com/home/Book/Data%20for%20website/Chapter8/Relatedness accessed on 28 September 2021.

This folder contains laboratory data from derived samples of three person mixtures using the ‘PowerPlex^®^ Fusion 6C’ kit and Dutch database frequencies from a study by Benschop et al. [73]. To explore whether closely related individuals will give a high *LR* when Mr X is substituted by a sibling, we specify following propositions:

*H*_1_: The DNA is from Mr. X

*H*_2_: The DNA is from an unknown contributor

A total of 100 siblings were simulated. The majority provide a low *LR* (exclusionary: *LR* < 1). A total of six *LR*s were greater than 100, with two approximating log_10_*LR* ≈ 6. However, if the propositions are altered to:

*H*_1_: The DNA is from Mr. X

*H*_2_: The DNA is from a sibling of Mr. X,

both *LR*s returned values less than one, favouring *H*_2_. This exercise illustrated that (a) close relatives can occasionally provide high *LR*s when tested against the proposition of unrelatedness, but (b) if the proposition is altered to ask the question of relatedness, then the evidence can support *H*_2_. This illustrates the importance of asking the right questions based upon the case-circumstances, i.e., when propositions are formulated, they must be reasonable and they must above all be based upon a clear understanding of the case circumstances.

#### 3.3.4. Deconvolution 

Deconvolution is used to predict the genotype of an ‘unknown’ contributor to a crime stain and it is typically undertaken to extract a profile in order to search a national DNA database. The method is described by Gill et al. [62], chapter 8.5.12, or in Section 4.3.1 for the specifics of the deconvolution model in *STRmix™*. There are several different ways to represent the data. The most common usage is to provide the ‘top marginal’ where the most likely genotype (for the unknown component) is extracted. Each genotype (per locus) is accompanied by ‘the ratio to next genotype’ which is the ratio of the top probability to the second highest probability. The larger the ratio, the greater the confidence in the genotype selected [50,131].

### 3.4. Investigative Forensic Genetics

#### Probabilistic Genotyping to Carry out Searches of National DNA Databases

*SmartRank* is based upon *LRmix Studio* [22,23], but was modified to enable searches of very large national DNA databases. A validation study [22] tested anonymised parts of the national DNA databases of Belgium, the Netherlands, Italy, France and Spain, along with a simulated DNA database. To each of the databases, 44 reference profiles were added. A total of 343 mixed DNA profiles were prepared from the reference samples, to act as the test set of data. Finally, the data were searched with both *SmartRank* and CODIS software.

Searches are most successfully employed when the mixtures are simple (major/minor) coupled with low levels of dropout. CODIS works by applying simple allele matching criteria whereas *SmartRank* takes account of allele drop-out, and was shown to be a more effective method to identify contributors for mixed profiles with low to moderate drop-out. *SmartRank* can be downloaded from https://github.com/smartrank/smartrank accessed on 28 September 2021 along with user guides; exercises are available at: https://www.dropbox.com/home/Book/Data%20for%20website/Chapter%2011/SmartRank_Exercises and chapter accessed on 28 September 2021 [62].

DNAmatch2 and CaseSolver are search engines which also adopt the quantitative model from *EuroForMix* [21,24]. A stepwise strategy is employed to search for matches, since a search using *EuroForMix* alone would be time-consuming. Consequently, the comparisons are filtered in a stepwise procedure. First, a simple matching allele count is carried out where for example, samples exceeding a defined drop-out level are rejected. The remaining comparisons are then searched using the qualitative *LRmix* model from *forensim* (similar to *SmartRank*). This step is very fast: samples providing *LR*s above a certain threshold are then re-tested using the quantitative *EuroForMix* model to provide a final list of ranked *LR*s. Studies show that quantitative models out-perform qualitative models [50,132]. *DNAmatch2* is used both as a database search engine as well as providing a platform to carry out contamination searches during routine casework, whereas *CaseSolver* is mainly used for profile comparisons in casework. Importantly, both *CaseSolver* and *EuroForMix* can conduct (reference to evidence) database searches; with the main difference that *CaseSolver* can perform this with many evidence items at the same time, and it provides a more flexible interface for data integration.

**Figure 4:**
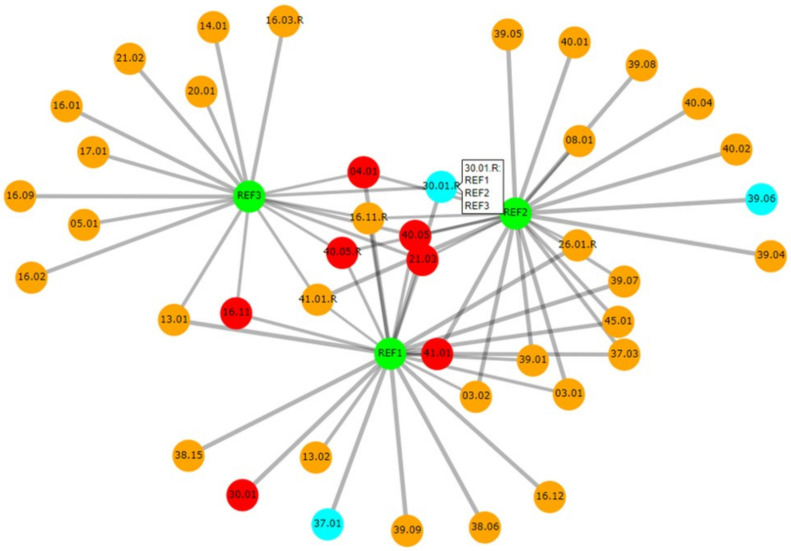
Graphical network summarizing the connections between case samples through *LR* calculations. The references are the green nodes. Single contributor evidences are in cyan; two contributors are in orange and three or more contributors are in red. If the ’plotly’ function in R is used then the mouse can be hovered over a node and this displays a list of the matches, as shown for sample 30.01. The thickness of the edges between the nodes is inversely proportional to the size of the *LR* on a log10 scale. Reprinted from [62], chapter 11, Copyright (2020) with permission from Elsevier.

*Casesolver* contains more functionalities than *DNAmatch2*, with the focus of being an effective and simple-to-use comparison tool for case officers (similar as *DNAxs)*. This software is especially designed to cope with complex cases which have a large number of evidence profiles and multiple reference samples. An example with 119 evidence profiles and three references is described by Bleka et al. [21]. *CaseSolver* compares each reference sample with each evidence profile, identifying potential ‘matches’ qualified by an *LR*. The second step carries out cross-comparisons between case-stains to identify unknown contributors. These can be deconvolved and used in further searches as required. If it is known that contributors may be related to each other, then simple relatedness searches can also be carried out. *CaseSolver* offers various ways to visualise or export the data, even to a comprehensive report; for example, an informative graphical network can be displayed that summarises the connections between the case samples (Figure 4). The latest version of *CaseSolver* (v1.8) provides a weight-of-evidence module which offers conservative corrections of *LR* for evaluative purposes, and automated report generation.

CaseSolver is available at: http://www.euroformix.com/casesolver accessed on 28 September 2021. Data and presentations are available at https://sites.google.com/view/dnabook/chapter-11?authuser=0 accessed on 28 September 2021.

### 3.5. Massively Parallel Sequencing (MPS)

Massive Parallel sequencing (MPS) is becoming increasingly used throughout the forensic community and may eventually supersede classic capillary gel (CE) methods [133]. MPS returns the entire sequence of a locus, not only the repeat region, but the flanking sequence as well; there is much more information to deal with compared to the standard repeat unit count used in classic CE. The main advantage of MPS is the potential to combine many more loci in multiplexes compared to CE. This results in much higher discriminating power. Shorter amplicon lengths should mean that more highly degraded DNA may be detected, but this will increase the potential to detect background DNA, as well as contamination. An additional challenge is that interpretation systems must be able to deal with profiles that are complicated by the presence of complex stutters.

Just and Irwin [134] developed a method of nomenclature of MPS-STRs that was based upon the longest uninterrupted sequence (LUS) and they used *LRmix Studio* to analyse mixtures. Later, the LUS nomenclature was extended to LUS+ [135], which is similar to that of Vilsen et al. [136], in order to identify as many different sequences as possible. They were able to identify 1050 out of 1059 sequences alleles. This system was adopted by Bleka et al. [137,138,139] who extended the analysis to the quantitative *EuroForMix* model. Instead of peak height (rfu), coverage (reads) are used to quantify allelic sequences. CE and MPS stutters are comparable [140]; ‘*n −* 1’ stutters are the most common to be found, but ‘*n* − 1’ and ‘*n* + 2’ forms are also observed, though the latter have much lower coverage and can be removed by filtering. Stutters can arise from different blocks of sequences within the same allele. Software packages such as *FDSTools* [141] are able to predict stutters, both simple and complex, based upon the allelic sequence.

The *EuroForMix* implementation of MPS-STR interpretation is described by [138,139] and both ‘*n −* 1’ and ‘*n* + 1’ stutters are accommodated from version 3. In order to obtain data in LUS/LUS+ format, the R program *seq2lus* can be used to convert raw sequence data derived from the ForenSeq Verogen Universal Analysis (UAS) software: https://verogen.com/wp-content/uploads/2018/08/ForenSeq-Univ-Analysis-SW-Guide-VD2018007-A.pdf accessed on 28 September 2021. To carry out the conversion, a look-up table file is used: Table S5 from Just et al. [135]. Once the nomenclature conversions are made, the analysis can proceed. The tool and updated look-up files, together with a tutorial is provided at: http://euroformix.com/seq2lus accessed on 28 September 2021. A more general tool called lusSTR, written in python, has been developed to avoid the need of a lookup table (available at: https://github.com/bioforensics/lusSTR accessed on 28 September 2021). 

Bleka et al. [138] explored the information gain, i.e., the LR increase, of the LUS vs. standard repeat unit (RU) nomenclature. Full profiles with the RU nomenclature provided an average log10LR = 37.04 whereas the LUS nomenclature returned log10LR = 43.3; the ratio is the theoretical information gain TIGRU→LUS = 1.17. However, the LRs are massive, and represent redundant information. Huge likelihood ratios have no benefit when presented in court. In practice any log10LR > 9 may be considered as providing redundant information because a greater LR has no impact upon a jury decision. Some jurisdictions e.g., UK have a reporting limit, upper threshold of 1 billion. 

Therefore, the main benefit of MPS-STR is related to the analysis of low-level DNA profiles that may be highly degraded, so that the probability of successful amplification is low. If the number of loci is increased, then the chance of successful amplification of a given locus is also increased and this will be reflected in an expected increased *LR* (provided that *H*_1_ is true). Doubling the number of loci from 27 loci to 54 loci will have an approximate proportionate doubling effect on the *LR* (log-scale). E.g., if log10*LR* = 2 for the former, it will return log10*LR* = 4 for the latter; if 128 loci are utilised then log10*LR* = 8, i.e., the more loci that are analysed, the more likely it is that reportable profiles can be achieved. We can summarise that the main advantage of MPS is the possibility to greatly increase the number of loci in the multiplex, the increased discrimination power per locus is secondary to this. 

In addition, Benschop et al. [142] examined allele detection and *LR*s obtained from STR profiles generated by two different MPS systems that were analyzed with different settings. The *LR* results for the over 2000 sets of propositions were affected by the variation for the number of markers and analysis settings used in the three approaches tested. Nevertheless, trends for true and non-contributors, effects of replicates, assigned number of contributors, and model validation results were comparable for the different MPS approaches and were similar to the trends observed in CE data. 

Even though sequence information from MPS technology provides higher data resolution, there is still a limitation in how mixture profiles, including major/minor components, are exported from MPS software. Two papers [138,142] point out that default analysis settings such as dynamic threshold potentially removes useful information forwarded for interpretation, weakening the ability to detect low-template components.

The above mentioned studies [142] demonstrate that probabilistic interpretation of MPS-STR data using the γ model in *EuroForMix* and *DNAStatistX* is fit for forensic DNA casework.

Probabilistic genotyping is not restricted to STRs, SNPs are also amenable [143,144]. Whereas STRs are multi-allelic, SNPs are generally di-allelic. This represents a particular challenge to assess the numbers of contributors because, with a maximum of two alleles in a population, we cannot use allele counting methods to ascertain this value.

Using a panel of 134 SNPs from Life Technologies’ HID-Ion AmpliSeq™ Identity Panel v2.2: https://www.thermofisher.com/content/dam/LifeTech/Documents/PDFs/HID-Ion-AmpliSeq-Identity-Panel-Flyer.pdf accessed on 28 September 2021, Bleka et al. [143] compared the *LRmix* model with *EuroForMix* showing that the latter was much more efficient especially when there are more than two contributors. The effective NOC is decided by following exploratory data analysis, outlined for STRs in Section 2.5, where the likelihood is maximised under *H*_2_. *LR*s obtained from overestimation of the actual NOC showed concordance with results compared to the actual NOC (from simulations up to six contributors). With the SNP panel tested, there is a limitation of that the mixture proportion (*M_x_*) of the POI must exceed 0.2 in order to achieve an *LR* > 100, although this restriction would be removed with much larger SNP panels. More recently, the performance of *EuroForMix* was compared to machine learning approaches [145]. 

The data used in the MPS SNP and STR publications cited, along with presentations available online: https://sites.google.com/view/dnabook/chapter-13?authuser=0 accessed on 28 September 2021.

### 3.6. Validation, Guidelines for Best Practice and Quality 

Developmental and internal validation of the probabilistic genotyping software *LRmix*, *LRmix Studio*, *SmartRank*, *EuroForMix*, *CaseSolver*, *DNAmatch2*, and *DNAxs/DNAStatistX* is described in internal validation documents; much information has been published [22,23,62,113,128]. Furthermore, there has been much research effort to gain insights into trends and to characterize the various models, as well as to inform guidelines for best practice. 

Using the qualitative model *LRmix Studio* research was carried out to show the effects of over- or under-assigning the NOC; the number of PCR replicates; the amount of DNA; and the drop-in rate [68,69,146,147,148].

The *SmartRank* output was compared to that of *LRmix Studio* in order to gain insight into the effects of model adaptations that enabled fast and efficient searching of voluminous databases [23]. In addition, the software was characterized in terms of the retrieval of true and non-donors; the effects of the size and composition of the DNA database; the number of contributors; the number of markers; and the level of drop-out [22,23]. As expected, positive effects on the retrieval of true donors were observed with: (1) a higher number of loci, (2) fewer contributors, (3) lower drop-out rates and/or (4) a higher discriminatory power. Retrieval of true donors was not influenced by the size of the DNA-databases used in this study (37,000–1.55 million). The size of the DNA-database, however, can have an effect on the retrieval of non-donors because of adventitious matches.

*LR*s generated from *EuroForMix* and *LRmix* were compared for true and non-donors to two- or three-person NGM DNA profiles [50] and to two- to four-person PPF6C DNA profiles [73]. This research demonstrated the effects of the NOC, over- or under-assigning the NOC; the number of PCR replicates; the amount of DNA; the level of unseen alleles for the person of interest; and the effect of increased PCR cycles. *H*_1_-true tests and *H*_2_-true tests were utilised. In the *H***_2_**-true tests, non-contributors were selected deliberately to a have large overlap with the alleles within the mixture and worst-case scenarios were examined where a simulated relative of one of the true donors was considered as the person of interest under the prosecution hypothesis [73]. A somewhat similar study was performed to compare MPS with CE-based DNA profiling data [142]. It was observed that the MPS read counts behaved in a similar manner to CE peak heights, and therefore similar results were obtained.

To summarize, the following overall trends were observed for CE and MPS profiles (note that exceptions can occur):

The lower the NOC and the lower the drop-out rate for the POI, the more often larger *LR*s were obtained.

The more donors and the more drop-out for the person of interest, the more often false-negative support was observed.

Using a lower NOC than designed yielded either equal results (predominantly with a true major donor as POI) or lower *LR*s. 

Over assigning the NOC hardly affected *LR*s for true major donors. 

An over-assigned NOC for *H*_2_-true tests can have the effect of increasing the *LR* to around neutral evidence.

False-positive support, *LR* > 1, was observed more often and with larger *LR*s when the POI was a (simulated) relative of a true donor rather than if the POI was an unrelated non-donor to the DNA mixture.

The use of multiple, instead of one PCR replicate, often increased the *LR* for true minor donors and decreased the *LR* for non-donors.

Based on the outcomes of the above-mentioned research studies, guidelines for best practice in forensic casework were developed. For *LRmix (Studio)* this included the exploratory data analysis approach, in which the effect of model parameters (such as the probability of drop-out) on the *LR* is examined. Non-contributor tests provide an indication of the range of *LR*s obtained if *H*_2_ is true. Although it is not mandatory, laboratories may find this a useful feature that can help to explain results in court. For *EuroForMix* and *DNAStatistX*, it is advised to perform model selection, to examine the model validation (‘pp-plots’) and iteration results and to report the *LR* only if defined criteria are met. Furthermore, for reasons of quality, efficiency and usefulness of performing weight of evidence calculations, guidelines for application of the *LR* models were developed by NFI. For *EuroForMix/DNAStatistX* these are presented in [128]; for instance there is an upper limit on the number of unseen alleles that a person of interest can have before an *LR* calculation is advised. Another quality aspect that relates to the use of the *DNAxs/DNAStatistX* software is the audit trail which automatically keeps track of who performed which action and when. 

Apart from software validation, guidelines for best practice and an audit trail, NFI invests in (automated) software testing during development, prior to release and during validation. This is important to ensure that the software is robust and behaves as designed. With a growing number of features, software testing becomes a very time-consuming task if performed manually. To save time, improve the test coverage, increase ad hoc and exploratory testing, and, in the end, reduce costs and maintenance, automated tests were designed and built for *DNAxs*. In the first three years after implementation of the *DNAxs* software suite, a total of 521 bugs were reported by the software engineers during development, by testers during validation, by users in casework, or by users performing research. Software bugs are errors, flaws or faults causing the software to produce an incorrect or an unexpected result, or to behave in unintended ways. The majority of bugs were solved in major or minor software releases that were planned and a minority required the release of a bug fix version or occurred during the development of these version. This shows that bug detection and debugging is part of the developmental and validation process, but also occurs in validated and released software versions. The reported bugs are viewed from a software perspective and relate to the use of the software or functionalities thereof. The observed bugs did not have effects on the DNA profile interpretation and/or reported conclusions. Further information on code coverage by testing, bug detection and debugging, but also information on the use of *DNAxs* (including *DNAStatistX* and *SmartRank*) and post-analytical errors in forensic casework can be found in [149]. Further details on the process of software testing can be found in [62], chapter 10. 

## 4. STRmix™

### 4.1. History of STRmix™ Creation

*STRmix™* is an Australian and New Zealand initiative that was jointly developed by Duncan Taylor from Forensic Science SA (FSSA) in Australia and John Buckleton and Jo-Anne Bright from the Institute of Environmental Science and Research (ESR) in New Zealand. *STRmix™* was first introduced into casework at FSSA and ESR in August 2012, however the events that lead to its development occurred three years prior.

Prior to 2010 there was no focussed effort to drive standardisation in forensic biology between laboratories in Australasia (Australia and New Zealand). Each laboratory had accrued knowledge and developed policies in a siloed manner, which meant that in one state Random Man Not Excluded (RMNE) also known as Cumulative Probability of Inclusion (CPI) was being used, in others likelihood ratios (*LR*), and amongst those there was a variety of implementations. In 2009 the Victoria Police Forensic Science Laboratory (VPFSL) in Melbourne had been using a software program called *DNAmix* [150,151] for calculating *LR*s in situations where unresolvable mixtures were obtained. *DNAmix* had been created as a result of DNA profile evaluations in the OJ Simpson trial, and the models within *DNAmix* required that no dropout-out had occurred. The VPFSL came to realise that this assumption was not being met in the evaluations being carried out, which resulted in the DNA profile evaluations in a number of cases being redone and reports reissued. One result in particular, which shifted an *LR* from 550 billion to 3, concerned Victoria’s police chief Simon Overland who ordered all DNA evidence be banned from court proceedings.

Following the laboratory shutdown, crisis talks were held with members of government forensic laboratories from across Australia and New Zealand. One of the outcomes was to form an Australasian forensic biology statistics working group in 2010 (with members from all government forensic laboratories from across Australia and New Zealand) with the overarching remit of standardisation across Australasia through the adoption of world leading evaluation and statistical practices. In this group were John Buckleton and Duncan Taylor, who started working on the ideas that would eventually become *STRmix™* (Taylor and Buckleton thankfully acknowledge the technical input of David Balding and the vision of Ross Vining, Linzi Wilson-Wilde and Keith Bedford). In 2011 Buckleton and Taylor presented the idea of *STRmix™* (initially to be called *DyNAmix*) to their member organisations, and the National Institute of Forensic Sciences (NIFS), and development was supported. Jo-Anne Bright joined the development team and by 2012 Taylor, Buckleton and Bright had completed development and validation on version 1.0 of *STRmix™*.

### 4.2. Probabilistic Genotyping and STRmix™

*STRmix™* considers parameters that describe some observed fluorescent peak collectively as ‘mass parameters’, ***M***. In essence probability of the observed data, given a genotype, treats these mass parameters as nuisance variables that are integrated across:

$$w_{j}=Pr(O|S_{j})=\int p(O,M|S_{j})p\left(M\right)dM$$

which assumes $p(M|S_{j})=p\left(M\right)$.

In *STRmix™* this integration is carried out using Markov Chain Monte Carlo (MCMC) sampling. The equation above applies across the whole profile. The locus terms (a superscript ‘*l*’) are a product within the integral, across all loci in the profile with data. The model of *STRmix™* then makes the assumption that the profile weight is approximated by the product of the integrals at each locus:

$$w_{j}=\int \underset{l}{\prod }p(O^{l},M|S_{j}^{l})p\left(M\right)dM=\underset{l}{\prod }\int p(O^{l},M|S_{j}^{l})p\left(M\right)dM$$

When tested, this assumption appears reasonable (see Figure 2 of [15]). As well as using weights to calculate the *LR*, having the weights themselves allows probing of the DNA profile data in powerful investigative ways, which we describe later.

Assumption 1 fulfils Slooten’s [152] requirement that “*we will assume that the model … parameters are chosen independently of the hypotheses.*” This allows the statement that the “*model cannot overstate the evidence very strongly very often for actual contributors: it cannot, averaged over all mixtures and contributors, happen with probability more than 1/t that the evidence is overstated by a factor t*.” 

We round out this claim by repeating Slooten’s [152] statement in his Equation (5.2), which has a pedigree back to Turing (quoted in Good [153]), that the probability of obtaining an *LR* ≥ *t* from a non-donor is $\leq \frac{1}{t}$.

### 4.3. Capabilities of STRmix™

#### 4.3.1. Deconvolution

The process of deconvolution is the assignment of weights to genotype sets. In other words, combinations of genotypes that could describe a profile(s) are considered, and a probability is assigned to them, proportional to how well they explain the observed peaks. In *STRmix™* this is achieved by integrating across a set of mass parameters using MCMC. The base model for *STRmix™* was described by Taylor et al. [15] and included parameters:

1. A template amount for each of the *n* contributors,
2. A degradation (described in [154]) which models the decay with respect to molecular weight (*m*) in the template for each of the contributors,
3. Amplification efficiency at each locus to allow for the observed amplification levels of each locus,
4. Replicate multipliers, which scales all peaks up or down between PCR replicates.

Later, Taylor et al. [155] extended the model to not only consider PCR replicates produced under the same system, but also DNA profiles from the sample produced under different conditions, i.e.,

Using the same DNA profiling kit, but with different laboratory processes (such as different PCR cycles or different models of laboratory hardware), 

Using two different DNA profiling kits 

which require the addition of mass parameter(s):

Kit multipliers, which scale all peaks in all replicates up or down between kits.

The expansion of the model for multiple kits/processes allowed various parameter freedoms between profiles produced by different processes. This was particularly useful for cold-cases where *STRmix™* could be used to combine the original profiling work with contemporary work in situations where technology had spanned multiple generations of profiling kit, and the DNA sample itself may have exhibited different degradation patterns between the generation of the two profiles.

Using a chosen set of values for the mass parameters allows the calculation of Total Allelic Product (TAP) [156], the total amount of fluorescence expected resulting from the amplification of an allele present in a DNA extract. As the PCR occurs, some of the fluorescence that was destined for the allele will shift to stutter positions on the EPG. The amount of the TAP that is expected to become stutter is based on the expected stutter ratios for each allele at each locus, either measured directly or regressed using the longest uninterrupted sequence (LUS) [156], or incorporating multiple repeat sequences of an interrupted STR in a multi-LUS model [136,157]. In reality, there are a number of stutter types that can occur, but the original *STRmix™* model (described in [15]) only included back stutter. This was later extended to include forward stutter (with modelling of forward stutter described in [157,158]), and then generalised so that any number of stutters (and with any size-based relationship to the parent peak), applied to any combination of specific loci, could be added by users with the mathematical framework automatically extending to incorporate them (generalised stutter modelling is described in [155]). The height of coincident peaks (either from multiple allele donations, or allele and stutter) are added [159,160] to produce the final expected peak heights.

The calculation of expected allele and stutter (of any combination thereof) peak heights using a set of mass parameters and expected stutter ratios ultimately results in a set of expected peaks and their heights, for each locus, in each PCR replicate and for each process or kit in which they have been generated. How well those mass parameter values explain the observed peak heights (or technically the probability of the observed data given the mass parameter values), depends on how well the observed and expected peak heights align.

Differences between an observed (*O*) and an expected (*E*) peak height are assigned a probability based on empirical models. These models take account of the magnitude of the difference and use a proxy for template to assess how tolerable such differences are. In order to determine how much to penalise stochastic effects in different types of fluorescence (i.e., allele, or stutter, or a combination), peak height variability models are used. An observed peak comprising a single fluorescence type, with peak height variability parameter value *c*^2^ is modelled by a log-Normal distribution with mean and variance parameters:

$$O\sim LN\left(ln(E),\frac{c^{2}}{f\left(E\right)}\right)\;\mathrm{where}\;f\left(E\right)=\frac{b}{E}+E$$

and *b* is a constant that typically takes values around 1000. The variance is inversely proportional to the function of expected peak height (which is approximately equal to E when E is large), which describes the well-known phenomenon that the relative size of stochastic peak height imbalance tends to increase as peak height decrease. The function *f*(*E*), deals with the fact that as the expected height decreases to very low levels the peak height variability starts to contract (see [161]). The constant *b* can be altered by the user from 0 (which turns off this low-level peak height variability contraction effect) to any arbitrarily high value. 

A range of distributions were investigated for modelling stutter in Bright et al. [162]. The log-normal appeared to fit the empirical data adequately.

When a peak comprises different fluorescence types, the sum of log-normal random variables is approximated by a shifted-log-normal distribution using moment matching. 

If a peak has an expected height, but is not observed, then it is modelled as a drop-out peak and a probability is used that is based on the integral of the probability of observing a peak between baseline and AT for a peak at expected height E. If a peak is not expected, but observed in the profile then it is modelled as a drop-in and a probability applied, based on the model of Puch-Solis [13].

The above process describes the generation of a set of expected peak heights and the probability for deviations of the expected heights from observed heights. The constant, ‘*b*’ in the variance term and the prior distribution parameters for *c*^2^ are set during model calibration in a tool called ‘Model Maker’ (described in Section 4.4). The MCMC process within *STRmix™* starts by assigning starting positions for all mass parameters, and randomly assigning genotype sets to each locus. This is the current set of parameters. Then, iteratively

(1) Choose a locus at random and propose a genotype set at that locus.
(2) Choose new values for all mass parameters by stepping a small distance from the values in the current set (known as a random walk, and with step size dictated by a Gaussian distribution). Propose these values.
(3) Calculate the expected peak heights using the proposed sample values.
(4) Calculate the likelihood value of the proposed sample values.
(5) Use a Metropolis-Hastings algorithm to accept or reject the proposed sample. If the proposed sample is accepted, then the proposed set of parameter values becomes the current set. If the proposed sample is rejected, then the proposal is discarded.
(6) Repeat steps 1 to 6 until a defined number of proposal accepts have been attained.

The first iterations of the MCMC are considered burn-in (the number of steps is set by the user) and discarded. After the burn-in the proportion of total iterations that a genotype set was the focus of the MCMC (called residence time) is the weight assigned to that genotype set.

A contributor that is assumed to be present under both *H*_1_ and *H*_2_ is described as a conditioning contributor. It is highly desirable to include as many contributors as possible as conditioning profiles. This is for practical reasons such as improving run time, but also for the much more important reason that any correctly assumed donor improves the ability of the system to differentiate between true and false donors [163]. Historically the use of conditioning contributors has been restricted to those situations where a donor is certain to correctly assumed, largely intimate samples. This underutilises the tool and is not in the interests of an innocent person accused of being a donor [95]. We recommend the extension of the use of conditioning to other evidential items such as assuming the presence of the habitual wearer of clothing and to any co-accused with a very high LR, especially if the POI is related to these persons [95,96]. Within a deconvolution, any number (up to the NOC from which the profile is designated as having originated) can be assumed as a conditioning profile, which during MCMC, locks their genotypes (known from their references) into any genotype set being considered.

Since *STRmix™* V2.6 the analysis of a profile can proceed without a set single number assigned for a profile as *STRmix™* has the ability to accept a range of contributors as the user input [164]. A range of contributors may be chosen if the user is unable to assign a single value due to the complexity or quality of the profile, or if different numbers may be required by the different parties in order for them to have their most probable explanations for the profile(s) [95,97]. The method works by carrying out a deconvolution of the evidence profile(s) given each NOC in the chosen range and then calculating the Bayes Factor (based on a method of Weinberg et al. [165,166]) to allow combination or contrast between the deconvolution. This is an alternative to the recent method described by Slooten et al. [94], but yields the same results under the same assumptions [71].

#### 4.3.2. LR Calculation

Given the weights from a deconvolution, the calculation of an *LR* in *STRmix™* is achieved by taking the ratio of the weighted sum of genotype set probabilities given two competing propositions. One or more references (some of which can have been assumed at the point deconvolution) are compared to the evidence profile(s) using propositions that are aligned with prosecution and a sensible alternative, in a way that best represents the strength of the evidence given the framework of circumstances of the case (see [3,71,95,103,167] for guidance documents on proposition setting). *STRmix™* can compare individuals to specific combinations of contributor positions within the evidence profile(s), generating an *LR* considering sub-sub-source level propositions [104], or to the mixture as a whole (i.e., all components and all combinations) to generate an *LR* that considers sub-source level propositions. When considering sources of DNA under the *H*_2_, *STRmix™* can consider these to be unrelated to the POI or related to the POI [168] in various relationship types. 

Following Balding [169,170] the different potential relationship types that the alternate donor could be to the POI can be assembled into one statistic. Writing$\;w_{j}=Pr(O|S_{j})$ and introducing the different relationship types *R_i_*, *i* = 1, *r*, where is the number of relationship types considered we obtain

$$LR=\frac{\sum_{j=1}^{J}w_{j}Pr(S_{j}|H_{1})}{\sum_{i=1}^{r}Pr(R_{i}|H_{2})\sum_{j=1}^{J}w_{j}Pr(S_{j}|H_{2},R_{i})}$$

The terms, $Pr(R_{i}|H_{2})$, represent the prior probability that a person related to the POI by relationship *R_i_* is the donor (given that the POI is not, *H*_2_). The expected prior proportions that different relative types can make up in the population, $Pr(R_{i}|H_{2})$, can either be set manually in *STRmix™* or estimated using the number of individuals in the population and the average number of children per family in that population [171].

*STRmix™* uses the sub-population model of Balding et al. [172], although the co-ancestry coefficient (*F_ST_*, θ) can be set to 0 by the user if desired in order to revert the calculation to using the ‘product rule’. 

When assigning probabilities for an allele in the population the value is set using a Dirichlet distribution with a uniform prior. The posterior means of the allele probabilities are then obtained by updating the prior with the counts of alleles in a database (using a similar method as described in [173]). For allele ‘*A*’, the probability of occurrence in the population, given it has been seen *x_A_* times out of *N* alleles is calculated by:

$$Pr(A)=\frac{x_{A}+k^{-1}}{N+1}\mathrm{in}\;\mathrm{early}\;\mathrm{STRmix}™\mathrm{versions}\;\mathrm{or}\;Pr(A)=\frac{x_{A}+{\left(k+1\right)}^{-1}}{N+1}\;\mathrm{in}\;V2.8\;\mathrm{and}\;\mathrm{higher}$$

where *k*^−1^ is the prior allele probability [174], and *k* is the number of different allele states that have been observed in the population. 

Multiple populations can be set up in *STRmix™*, each with their own allele frequency file, θ and population proportion in the local geographical region. This allows *STRmix™* to calculate an *LR* considering the contributors to be from any of the set populations individually, or anyone in the local geographical region, by stratifying across all populations [175].

In addition to the point estimate *LR STRmix™* also provides a credible interval [176] using the highest posterior density (HPD) method. While there has been justified debate over the provision of intervals for *LR*s [177,178,179,180,181,182,183,184], it is the preference for most practising forensic DNA laboratories to report a lower bound interval on the *LR* value. Within *STRmix™* the HPD interval optionally takes into account any combination of:

(1) sampling variation in allele frequency database
(2) sampling variation in the iterations of the MCMC leading to the assignment of weights
(3) uncertainty in the value of θ

The second of these factors intends to capture the amount of variability in the *LR* resulting from Monte Carlo resampling inherent within MCMC processes. More recently this process has been found to have variable coverage [185] although taken collectively with other aspects of conservative behaviours the coverage is good [186] The third of these factors is achieved by *STRmix™* being able to take a point value for θ or a distribution described by a β distribution (see [187,188,189] for examples of θ distribution models). 

The result of an *LR* calculation on *STRmix™* is that the user can be provided with the *LR*s for the:

- sub-sub-source proposition pair,
- the sub-source proposition pair considering the alternate DNA donor as
  ○ unrelated,
  ○ sibling,
  ○ half-sibling,
  ○ parent/child,
  ○ aunt/uncle/niece/nephew,
  ○ grandparent,
  ○ cousin, or
  ○ unified across all relationship types,
- all of the above for:
  ○ each ethnic population in the local geographical region, and
  ○ stratified across all populations,
- all of the above for:
  ○ each NOC in a chosen range, and
  ○ stratified across the range (or with bespoke NOC choice for proposition),

and all with an associated HPD interval to account for uncertainty in allele frequencies, weights and θ. As well as generating *LR*s for the comparison of a reference to a deconvolution, *STRmix™* can also compare a list of references in a database. Using an *LR* threshold provides a search capability [190]. This is not restricted to resolved genotypes of a single individual, but rather can be applied to any profile (single source or mixtures, resolved or unresolved). Using the *LR* calculation feature than considers a relative of a POI as a source of DNA, allows *STRmix™* to carry out familial searches against any profile (again from complete single source to complex and unresolved mixtures) [168]. This lead to Australia’s first conviction resulting from a familial search [191]. The ability to search any DNA profile against a database is also commonly used as a Quality Assurance tool to screen evidence profiles for potential contamination or assist in environmental monitoring [192].

*STRmix™* has a profile sampling tool that can be used to generate profiles from a population and calculate *LR*s by comparison to a deconvolution. These profiles can either be generated using probabilities based on their probability of occurrence in a population [59] or using importance sampling [60] in order to build distributions of ‘*H*_2_ true’ *LR*s and determine exceedance probabilities. Exceedance probabilities (sometimes called non-contributor performance tests [9]) provide the probability of randomly selecting someone from the population who would provide a particular level of support (typically for their inclusion to the profile, and typically the level of interest is that yielded by a *POI*). These probabilities, however, are fundamentally different from *LR*s and should not be confused with, or substituted for *LR*s in evaluations [193]. The LR itself provides an upper bound on exceedance probabilities (a simple proof was provided in [59] so that if LR_POI_ is produced by the comparison of a POI to an evidence profile the statement can be made:

*The probability of observing a likelihood ratio of LR_POI_ or larger from an unrelated non-donor is less than or equal to 1 in LR_POI_.*

Another database searching tool available in *STRmix™* is to carry out a top-down analysis [74] in which only the main contributors to a highly complex profile are compared. In concept, a top-down analysis is similar to carrying out an evaluation on a major ‘cluster’ of peaks in a profile [194]. The process works by carrying out deconvolutions in steps, where in each step the AT is altered (on a per-locus basis) and only the peak information that meets or exceed the step-AT is used. Each deconvolution considers the profile as originating from N individuals (where N here represents the top N contributors in which you are interested, to the profile and not the total number of contributors to the profile). At the conclusion of each step’s deconvolution the result is searched against a database and individuals that yield an LR that exceed a pre-determined LR threshold are flagged. The first step raises the AT (on a per-locus basis) to the height of the highest peaks and each step lowers the ATs by a proportion of the distance from the highest peak to the standard AT used. The steps stop when the profile can no longer be described by N individuals (due to excessive peaks that cannot be explained as allele, stutter or drop-in). The final database search report returns individuals flagged at any step, with the maximum LR attained across all steps in which they were flagged. This method has been extended to the quantitative model of *STRmix™* and has been trialed on a set of complex mixtures (5+ contributors) from the no-suspect workflow of a forensic laboratory, showing an 80% link rate with someone on the local database [195].

### 4.4. Implementation of STRmix™

**Figure 5:**
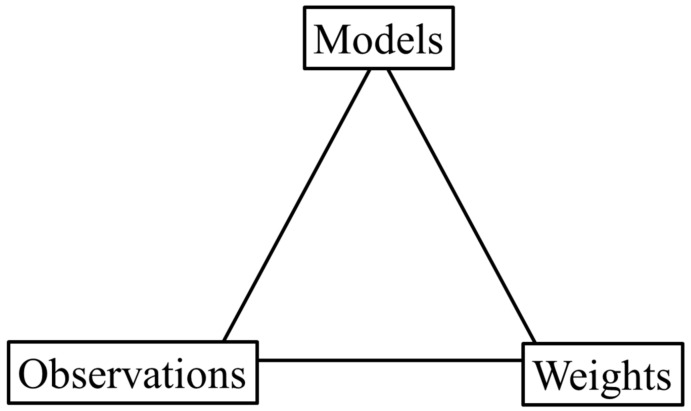
Representation of the components involved in the analysis of a DNA profile.

*STRmix™* utilises general models to describe DNA profile behaviour, for example that peak height variability increases as peak heights decrease, or that loci can vary in their amplification efficiencies depending on the PCR micro-environment, or that stutters are expected to occur at a particular size (relative to their parent peak size) and height (dependent on their parent peak height). Each of these models have parameters that can be calibrated to the performance of DNA profile generation in a particular laboratory or for a particular process. A standard objective for a DNA profile analysis is to use the calibrated models, and the observed data in order to obtain weights. Figure 5 shows these three aspects diagrammatically, each connected to the other two.

Using this relationship, if the weights are known for a set of observed profiles, then this information can be used to calibrate the models. Within *STRmix™* there is a feature known as Model Maker [196], which takes sets of single source input profiles (whose donor references are known) to calibrate parameters in the *STRmix™* models. The general process is carried out with a component-wise MCMC, whereby cycles of sampling mass parameters (on a per profile basis) followed by sampling of variance parameters (on a dataset wide basis) eventually reaches a steady-state for all parameters in the dataset. The variance parameter values are then attuned to the behaviour of profiles produced under the conditions tested and can be set in *STRmix™* as the default values for casework use.

Some, parameters such as expected stutter ratios have been shown to be robust to laboratory effects [197], while others are more sensitive [196]. The most important factors for peak height variability have been found to be the number of PCR cycles carried out during amplification, the PCR kit used and the models of electrophoresis instrumentation [196]. The use of prior distributions rather than point values for parameters such as variances with the calibration setting the hyper-parameters of the prior distribution (using hierarchical Bayesian modelling) has shown that general settings can be applied and are robust to a wide range of factors [198]. Despite this robustness, it is standard practice (and the recommendation of the *STRmix™* group, the SWGDAM 2015 [108] and the ASB guidelines (Standards for Validation Studies of DNA Mixtures, and Development and Verification of a Laboratory’s Mixture Interpretation Protocol: http://asb.aafs.org/ accessed on 28 September 2021)) that each laboratory implementing *STRmix™* undertakes a calibration prior to use on casework samples. In part this ensures the best alignment of the prior distributions for the input parameters to the validation data.

### 4.5. DBLR—A Companion Product to STRmix™

*STRmix™* has closely aligned companion product *DBLR* (which stands for DataBase Likelihood Ratio, https://www.strmix.com/dblr/ accessed on 28 September 2021), which can take the results of a deconvolution and carry out interrogations of the results. An overview and developmental validation is given by Kelly at el [199]. It also has the flexibility and power to construct propositions that consider aspects of relatedness of contributors within a sample or between samples (including common DNA donorship). *DBLR* has databasing properties that allow automated, and auditable searching of deconvoluted mixtures against a reference DNA database.

The investigative properties of *DBLR* allow the user to probe the deconvolution to see what DNA profiles are the most supported by the data, to gauge the discrimination power of the analysis by producing distributions of *LR*s expected for contributor and non-contributors [200], or to calculate exceedance probabilities.

Two mixtures can be compared using *DBLR* in order to determine whether there is support for a common DNA donor, using the method described by Slooten [20]. Studies into this feature have found a high efficacy for identifying common donors [159]. The process has been shown to have use as a Quality Assurance tool to identify potential sample to sample contamination events [25].

Recently *DBLR* has implemented a general framework for the comparison of deconvoluted profiles or references. This general framework allows multiple deconvolutions, from different evidence samples that are believed to possess a common DNA donor, to be considered together in order to obtain better resolution in the genotypes of the donors to any (or multiple) of the profiles [201]. The distribution of probabilities across a number of potential genotypes (or genotype sets) can be thought of in similar ways whether considering a mixture deconvolution, or a kinship calculation [152]. Using this idea, the general framework in *DBLR* allows the setting-up of competing pedigrees within the evaluation [202]. The pedigrees can be linked to components of mixtures so that bespoke propositions can be set up to consider issues such as:

- How many common donors are in a mixture?
- Are any donors of the multiple mixtures related (see [203] for an investigation into the effect of not recognising relatedness in mixtures)?
- If I assume a relative of a POI to one mixture does that assist in resolving the other components?
- If I use multiple mixed samples from a disaster victim identification (DVI) together in a single analysis will that help to better resolve the genotypes of the donors?

The variation in potential propositions is virtually limitless. These types of questions, and the evaluations that follow, have applications to standard casework, but also DVI, or investigations looking into serial offenders, or offences involving multiple family members.

### 4.6. Validation of STRmix™ 

When *STRmix™* was first implemented into active casework in 2012, probabilistic genotyping was not as prevalent as it is in 2021. There were not yet the standards developed as there are now for the validation or use of such software [108,109,204]. In these guidance documents there are outlined various requirements for the development, validation, and implementation of probabilistic genotyping systems. One of the criteria mentioned is that the statistical and biological models used be published or otherwise generally accessible. In Table 1 we provide a list of publications that detail the models used within *STRmix™*. 

The publications relating to *STRmix™* models (and some initial validation work) were initially all described in the Taylor et al. publication “*The interpretation of single source and mixed DNA profiles*” [15]. Since that publication, updates in models occurred over time and so by necessity have appeared in numerous publications. All information about modelling and validation are compiled in a single document in the *STRmix™* manuals, provided to users of the software and available to the defense under a non-disclosure agreement.

As well as detailing the machinations of the models, testing of their performance on data is required to show foundational validity and validity of application. There have been numerous such published validations of the performance of *STRmix™* modelling either in part or as a whole and we provide a list of these in Table 2. Many of these validations have come from the desire of the developers (and more recently users) of *STRmix™* to know the performance of the models, but some have come as responses to published perceptions of short-comings in validation efforts (for example the report of the President’s Council of Advisors on Science and Technology [117]). 

In a brief summary *STRmix™* publications on validation work from Table 2 include data from profiling kits; Profiler Plus, Identifiler Plus, Fusion (5C, 6C), NGM SElect, GlobalFiler, PowerPlex (21, ESI17 Pro, 16 HS, ESI17 Fast), SGMPlus and MiniFiler (we are also aware of numerous other profiling kits that have been validated for use in casework, but do not have the work published). There have been hundreds of millions of ‘*H*_2_ true’ tests, spanning over 3000 laboratory-produced profiles under a range of conditions (from 26 to 34 PCR cycles, and on multiple models of thermocycler or electrophoresis instrument) and of complexity spanning single source to six person mixtures.

### 4.7. Growth of STRmix™ 

**Figure 6:**
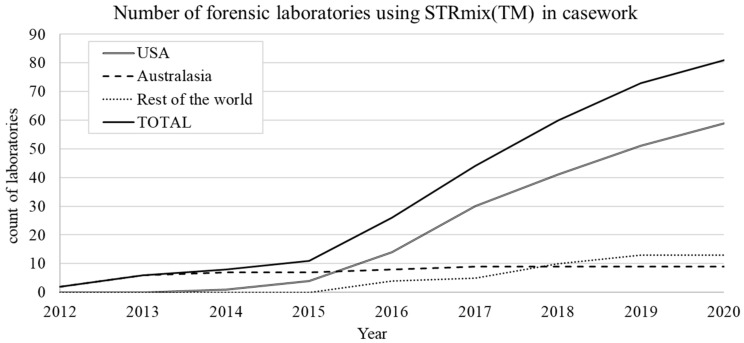
Growth of *STRmix*™ use over eight years.

Since its introduction in 2012 in ESR and FSSA, *STRmix™* has now been adopted throughout Australia and New Zealand and currently over 60 DNA forensic laboratories throughout the world, including the US army and the Federal Bureau of Investigation (FBI). Figure 6 shows the growth of *STRmix™* between 2012 and 2020. 

When last tallied, *STRmix™* is believed to have been used in over 220,000 cases worldwide, available online: https://www.strmix.com/news/survey-shows-strmix-has-been-used-in-220000-cases-worldwide/ accessed 28 September 2021. For the past 6 years, users in the USA have organised a yearly *STRmix™* workshop/conference, that had over 750 attending online presentations in 2020.

The *STRmix™* team has grown from an original three developers to employing over 20 individuals who work in training, support, validation, research and development, programming and quality assurance. 

### 4.8. Admissibility Experiences with STRmix™

The *STRmix™* group’s experience with court and admissibility is mainly in the USA and Australia. In the, at least, 83 admissibility hearings to which *STRmix™* has been subjected (as of 1 January 2021), a very wide range of issues have been raised but there are a few that recur. These can be divided into those specifically aimed at *STRmix™*, those aimed at PG in general, and those that relate more broadly to the use of *LR*s and especially understandability and the verbal scale. See information available online: https://johnbuckleton.wordpress.com/strmix/ accessed 28 September 2021, for a non-exhaustive list of admissibility hearings, most with attached rulings or transcripts

Recurrent issues that have been raised regarding *STRmix™* include independence of validation, run to run variability, code access, code quality, and validation.

#### 4.8.1. Independence of Validation

Perhaps the most recurrent complaint refers to the established fact that most publications on STRmix include one or more of the original developers. This is exemplified by two comments in the PCAST [117] report. PCAST @ pg 79 give: “*Appropriate evaluation of the proposed methods should consist of studies by multiple groups, not associated with the software developers, that investigate the performance and define the limitations of programs by testing them on a wide range of mixtures with different properties*.” PCAST @ pg 81 give: “*As noted above, such studies should be performed by or should include independent research groups not connected with the developers of the methods and with no stake in the outcome*.”

This argument has been taken further to suggest that even those labs separate from the developers but who have purchased *STRmix™* have a vested interest and hence their publications should not be trusted.

Whilst we reject the suggestion that we, and the many professional collaborators that we have worked with, would distort our publications because of self-interest we acknowledge the desire to have further distance between the validators and the developers. The National Institute of Standards and Technology (NIST) is often suggested as a suitable organization for this purpose [218]. NIST has had STRmix since March 2014 initially as an evaluation copy but subsequently purchased. There is one publication from NIST [217] that represents a comparison of *STRmix™* and *EuroForMix* and both software perform well. There is desire for more independent work to be done and the developers would like to assist, at an appropriate distance, any efforts at independent validation.

#### 4.8.2. Run to Run Variability

One complaint is that the MCMC means that the exact value reported for the *LR* could be different if the process were rerun. The argument is that the existence of variability raises doubts about whether any of the results should be accepted. This raises very significant questions about precision and accuracy that we will touch on briefly later. However, a full treatment is beyond the scope of this work. Here we simply note that *STRmix™* includes a partially successful attempt to give a lower bound to the MCMC variability [185]. In conjunction with other conservancies, it is almost certain that the *LR* is understated in the overwhelming majority of cases [186].

#### 4.8.3. Code Access

The code for open source software is freely available on the internet. *STRmix*’s code is not open source but is available under an non-disclosure agreement (NDA) [219] This meets the ISFG guidelines [109] requirement “*However, if requested by the legal system, the code should be made available subject to the software provider’s legitimate copyright or commercial interests being safeguarded. Supervised access to the code under a “no copy” policy is acceptable*.” Objections to the use of an NDA have included that inconvenience of supervision and the risk presented by the sanctions agreed to in the NDA if the NDA is contravened.

#### 4.8.4. Code Quality

The *STRmix™* code has been inspected three times by the same independent analyst under NDA. The comments made centre around coding practice, documentation, and adherence to software engineering standards [220]. At no stage has a coding fault been identified that affects the accuracy of the output although comments have been made that certain coding practices might increase the risk of, as yet undiscovered, miscodes. The *STRmix™* team maintain regularly updated specifications documentation, risk analysis, and a gap analysis. This latter specifies any gaps between current *STRmix* practice and various guidance documents. In summary *STRmix™* complies or very nearly complies with the SWGDAM [108], ISFG [109], Forensic Science Regulator [116] and IEEE requirements [221]. The *STRmix™* group is accredited to ISO 9001 standard.

#### 4.8.5. Validation

Various aspects of *STRmix™* validation have been challenged in courts since 2012. The initial challenges related to the conceptual validation of the models used by *STRmix™* or the laboratory in-house validation of the software to show that it was performing to a high standard. Later challenges moved to the thoroughness of developmental validation, the adherence of developmental validation with published guidelines, or the validation of the computer coding (separately from the validation of the results produced by the computer code). This latter point was the focus of a multiple day defence challenge to *STRmix™* in R v Tuite [222] in Australia.

## Figures and Tables

**Table 1:** Publications of conceptual components of *STRmix*™ modelling.

Algorithms, Scientific Principles and Methods	Version Introduced	Reference
Allele and stutter peak height variability as separate constants within the MCMC	V2.0	[15]
Peak height variability as random variables within the MCMC	V2.3	[196]
Model for calibrating laboratory peak height variability	V2.0	[196]
Application of a Gaussian random walk to the MCMC process	V2.3	[205]
Modelling of back stutter by regressing stutter ratio against allelic designation	V2.0	[156,197,206,207]
Modelling of back stutter by regressing stutter ratio against LUS	V2.3	[156,162,206,207]
Modelling of forward stutter	V2.4	[157]
Modelling of allelic drop-in using a simple exponential or uniform distribution	V2.0	[15]
Modelling of allelic drop-in using a γ distribution	V2.3	[13]
Modelling of degradation and dropout	V2.0	[154]
Modelling of the uncertainties in the allele frequencies using the HPD	V2.0	[208]
Modelling of the uncertainties in the MCMC	V2.3	[171,208,209]
Database searching of mixed DNA profiles	V2.0	[190]
Familial searching of mixed DNA profiles	V2.3	[168]
Relatives as alternate contributors under the defence proposition	V2.3	[168]
Modelling expected stutter peak heights in saturated data	V2.3	[157]
Taking into account the ‘factor of two’ in *LR* calculations	V2.3	[104]
Model for incorporating prior beliefs in mixture proportions	V2.3	[210]
Combining DNA profiles produced under different conditions into a single analysis	V2.5	[155]
Assigning a range for the number of contributors to a DNA profile	V2.6	[164]
Mixture-to-mixture comparison to identify common DNA donors	V2.7	[20]
A top-down DNA search approach	V2.8	[74]
The diagnostic outputs of *STRmix™*	V2.3	[211]

**Table 2:** Publications of validation of *STRmix*™ models.

Focus of Validation	Reference
Ability of *STRmix™* to deconvolute profiles and assign *LR*s that comport to manual interpretation and human expectation	[15]
Ability of *STRmix™* to discriminate between donors and non-donors in database searches	[190]
Behaviour of *STRmix™* to assign *LR*s when dealing with multiple replicates, different number of contributors, and assumed contributors	[163]
Sensitivity of *LR* produced by *STRmix™* to different factors of uncertainty such as theta, relatedness of alternate DNA source and length of MCMC analysis	[171]
Tests to be performed when validating probabilistic genotyping, using *STRmix™* as an example	[112]
Ability of individuals from different laboratories to standardise evaluations when using *STRmix™*	[33,53]
Ability of *STRmix™* to reliably use peak height information in very low intensity profiles	[56,132,210]
Ability of *STRmix™* to discriminate between donors and non-donors in large-scale Hd true tests, or using importance sampling	[59,60,190,200,212,213]
Sensitivity of *STRmix™* model parameters to laboratory factors	[196,198]
Ability of *STRmix™* to utilise information from profiles produced under different laboratory conditions within a single analysis	[155]
Effect of mixture complexity, allele sharing and contributor proportions on the ability *STRmix™* to distinguish contributors from non-contributors	[54]
The ability of *STRmix™* to identify common DNA donors in mixed samples	[25,159]
The sensitivity of *LR*s produced in *STRmix™* to the choice of the number of contributors	[71,72,97]
Ability to use *STRmix™* to resolve major components of mixtures	[72]
Testing the assumption of additivity of peak heights in *STRmix™* models	[159,160]
Performance of the degradation model within *STRmix™*	[214]
The effect of relatedness of contributors to the *STRmix™* analysis	[203,215]
Testing the calibration of *LR*s produced in *STRmix™*	[58]
Validation overviews of *STRmix™*	[205,216]
Comparison of *STRmix™* to other probabilistic genotyping software	[41,43,112,217]

## Supplementary Materials

The following are available online at https://www.mdpi.com/article/10.3390/genes12101559/s1, Supplement S1: Details of available software to carry out probabilistic genotyping, Supplement S2: Details of the gamma model.
