# Supplementary material for: A Review of Probabilistic Genotyping Systems: EuroForMix, DNAStatistX and STRmix™
Source: Genes (Basel). 2021 Sep 30;12(10):1559. doi: 10.3390/genes12101559 (PMC8535381; doi:10.3390/genes12101559)
Supplement: Supplementary file 1 [file genes-12-01559-s001.zip › genes-1330678-supplementary.pdf]

Supplement S1: Details of available software to carry out probabilistic genotyping

Software	Approach	License	Reference
CeesIT	Continuous	(a)	[1]
DNAmixtures	Continuous	Open-source[(b)]	[2]
DNAStatistX	Continuous	Open-source	[3]
DNA-View Mixture Solution	Continuous	Commercial	(c)
eDNA	Continuous	(d)	(d)
EuroForMix	Continuous	Open-source	[4]
Genoproof	Continuous	Commercial	[5]
Kongoh	Continuous	Open-source	[6]
Lab Retriever	Semi-continuous	Open-source	[7]
LikeLTD v.6	Continuous[(e)]	Open-source	[8]
LiRa	Semi-continuous/continuous [(f)]	Commercial	[9]
LoCIM-tool	Empirical	Open-source	[10]
LRmix/LRmix Studio	Semi-continuous	Open-source	[11]
MaSTR	Continuous	Commercial	[12]
STRmix^{TM}	Continuous	Commercial	[13]
TrueAllele	Continuous	Commercial	[14]

Table S1: A list of some of the available software, license conditions and source. Reprinted from Gill, P, Bleka, Ø, Hansson, O, Benschop, C and Haned, H (2020) Forensic Practitioner's Guide to the Interpretation of Complex DNA profiles, London, Academic Press, chapter 7, Copyright (2020) with permission from Elsevier.

a) Ceesit license is: "no cost to local, state and federal forensic DNA laboratories or entities pursuing research, forensic validation or education for non-commercial purposes", but a full license is required for commercial use.

b) DNAmixtures is a free of charge open-source R package, however it requires the HUGIN commercial software to run.

c) *DNA-View: http://dna-view.com/ and http://dna-view.com/downloads/Mixture%20Solution%20poster.pdf*
d) *eDNA: http://ednalims.com/probabilistic-genotyping/. Freely available web-based software available to consortium members. Bullet uses LRmix and Bulletproof uses EuroForMix. Each program has a custom built graphical user interface.*
e) *LikeLTD also has a semi-continuous model [15]*
f) *LiRaHT is available as a continuous model: https://cdnmedia.eurofins.com/europeanwest/media/1418957/lgc_lira_fact_sheet_en_0815_90.pdf*

Of the continuous models listed in Table S1, *DNAmixtures, DNAView Mixture Solution, DNAStatistX, LikeLTD, LiRaHT* and *EuroForMix* all use the gamma model (Supplement 2) for peak height, but there are differences between software assumptions. *CeesIT* uses a normal distribution. *Kongoh* estimates peak height distributions by using the Monte Carlo simulation based on experimental data to consider allele or locus-specific effects. *STRmix*^{TM} and *TrueAllele* are both commercial solutions, based on a Bayesian approach through specifying prior distributions on the unknown model parameters. They use Markov Chain Monte Carlo (MCMC) methods [16, 17] to calculate marginalised likelihood expressions by simultaneously sampling over the discrete set of genotypes for the unknown contributors specified in the model and the unknown parameters. *MaSTR* and *GenoProof Mixture* also employ MCMC in their calculations.

Supplement S2: Details of the gamma model

The gamma distribution has useful properties: the shape and scale parameters are calculated from M_x, the mixture proportion for contributor 1 and *1-Mx* for contributor 2; ω is the coefficient of peak height variation and μ is the peak height expectation.

The expected peak height of any given allele is obtained by multiplying together shape and scale parameters:

$$\mu = E[Y] = \alpha \times \beta \qquad (1)$$

The coefficient of variance (ω) is the standard deviation divided by the peak height expectation:

$$\omega = CV[Y] = \alpha^{-\frac{1}{2}} \quad (2)$$

Hence the shape and scale parameters are defined as:

$$\alpha = \frac{1}{\omega^2} \quad (3)$$

$$\beta = \mu\omega^2 \quad (4)$$

The per contributor parameters are calculated as follows:

- Contributor 1: $\alpha_1 = M_x/\omega^2$ (5)
- Contributor 2: $\alpha_2 = (1 - M_x)/\omega^2$ (6)
- $\beta = \mu \times \omega^2$ (7)
- $\mu_1 = \alpha_1 \times \beta$ (8)
- $\mu_2 = \alpha_2 \times \beta$ (9)

The gamma model calculates the values of the shape and scale parameters by maximum likelihood estimation. With this method, the value of the evidence under $Pr(O|H_1)$, by optimising the values of M_x, ω, μ . The values are adjusted via a large number of iterative steps, until the log likelihood value is maximised. The process is repeated to calculate the value of the evidence under $\Pr(O|H_2)$ to generate a second maximised log likelihood.

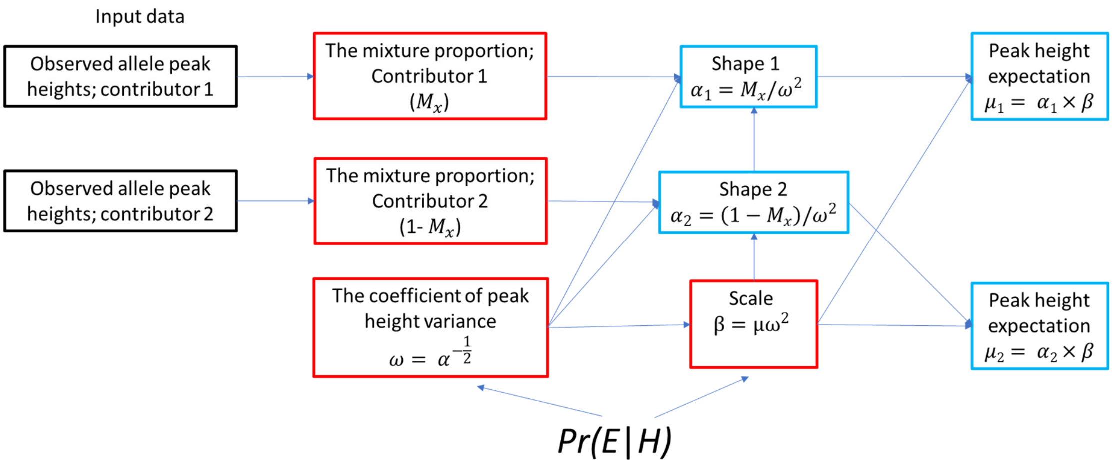

Fig. S1: An outline of the maximum likelihood estimation of the basic gamma model. The input data are the allele peak heights; the M_x, ω, μ parameters are estimated by optimisation (outlined in red): the values are iteratively adjusted to maximise the values of $\Pr(O|H_1)$ and

$\Pr(O|H_2)$ respectively, using separate parameter optimisations in order to derive the likelihood ratio.

References

1. Swaminathan, H., et al., *CEESIt: A computational tool for the interpretation of STR mixtures.* Forensic Sci Int Genet, 2016. **22**: p. 149-160.
2. Graversen, T. and S. Lauritzen, *Computational aspects of DNA mixture analysis.* Statistics and Computing, 2015. **25**(3): p. 527-541.
3. Benschop, C.C.G., et al., *DNAxs/DNAStatistX: Development and validation of a software suite for the data management and probabilistic interpretation of DNA profiles.* Forensic Sci Int Genet, 2019. **42**: p. 81-89.
4. Bleka, Ø., G. Storvik, and P. Gill, *EuroForMix: an open source software based on a continuous model to evaluate STR DNA profiles from a mixture of contributors with artefacts.* Forensic Science International: Genetics, 2016. **21**: p. 35-44.
5. Götz, F.M., et al., *GenoProof Mixture 3—New software and process to resolve complex DNA mixtures.* Forensic Science International: Genetics Supplement Series, 2017. **6**: p. e549-e551.
6. Manabe, S., et al., *Development and validation of open-source software for DNA mixture interpretation based on a quantitative continuous model.* PLoS One, 2017. **12**(11): p. e0188183.
7. Inman, K., et al., *Lab Retriever: a software tool for calculating likelihood ratios incorporating a probability of drop-out for forensic DNA profiles.* BMC Bioinformatics, 2015. **16**: p. 298.
8. Steele, C.D., M. Greenhalgh, and D.J. Balding, *Verifying likelihoods for low template DNA profiles using multiple replicates.* Forensic Sci Int Genet, 2014. **13**: p. 82-9.
9. Puch-Solis, R. and T. Clayton, *Evidential evaluation of DNA profiles using a discrete statistical model implemented in the DNA LiRa software.* Forensic Sci Int Genet, 2014. **11**: p. 220-8.
10. Benschop, C.C. and T. Sijen, *LoCIM-tool: An expert's assistant for inferring the major contributor's alleles in mixed consensus DNA profiles.* Forensic Sci Int Genet, 2014. **11**: p. 154-65.
11. Haned, H., K. Slooten, and P. Gill, *Exploratory data analysis for the interpretation of low template DNA mixtures.* Forensic Sci Int Genet, 2012. **6**(6): p. 762-74.
12. Adamowicz, M., et al., *Validation of MaSTR™ software: Extensive study of fully-continuous probabilistic mixture analysis using PowerPlex® Fusion 2–5 contributor mixtures.* Forensic Science International: Genetics Supplement Series, 2019. **7**(1): p. 641-643.
13. Taylor, D., J.A. Bright, and J. Buckleton, *The interpretation of single source and mixed DNA profiles.* Forensic Sci Int Genet, 2013. **7**(5): p. 516-28.
14. Perlin, M.W. and A. Sinelnikov, *An information gap in DNA evidence interpretation.* PLoS One, 2009. **4**(12): p. e8327.
15. Steele, C.D., M. Greenhalgh, and D.J. Balding, *Evaluation of low-template DNA profiles using peak heights.* Stat Appl Genet Mol Biol, 2016. **15**(5): p. 431-445.
16. Hastings, W.K., *Monte Carlo sampling methods using Markov chains and their applications.* 1970.
17. Metropolis, N., et al., *Equation of state calculations by fast computing machines.* The journal of chemical physics, 1953. **21**(6): p. 1087-1092.
